# Loss of the liver circadian clock affects the expression of intrarenal renin-angiotensin system components

**DOI:** 10.1038/s41598-025-34303-w

**Published:** 2025-12-29

**Authors:** Soha A. Hassan, Michael Stumpe, Ka Yi Hui, Marie-Nöelle Giraud, Urs Albrecht, Jürgen A. Ripperger

**Affiliations:** 1 Department of Biology, Faculty of Science and Medicine, University of Fribourg, Fribourg, 1700 Switzerland; 2 Department of Zoology, Faculty of Science, Suez University, Suez, 43518 Egypt; 3 Department of EMC, Faculty of Science and Medicine, University of Fribourg, Fribourg, 1700 Switzerland

**Keywords:** Blood pressure, Circadian clock, Clock genes, Intrarenal RAS, Liver, RAAS system, Diseases, Physiology

## Abstract

The intrarenal renin-angiotensin system (irRAS) regulates Na^+^ and fluid retention to counteract low blood pressure. Some of its components are expressed with daily rhythms. The circadian metabolism and physiology of the liver can synchronize gene expression in other organs. Hence, we tested whether the liver circadian oscillator could coordinate daily fluctuating expression of irRAS components. To this end, we used two different knock-out mouse strains lacking circadian oscillators specifically in hepatocytes to investigate the impact of this inactivation on their irRAS. Surprisingly, daily fluctuations in expression of many irRAS components in these animals were still detectable but with different phases. The data indicate that signals from liver can synchronize most of the irRAS components tested independent of its circadian oscillator. Both hepatocyte-specific knock-out mouse strains had reduced expression of the Na^+^/H^+^ exchanger 3 (*Nhe3*) in kidney, which may explain the ~ 25% reduction in their systolic and diastolic blood pressure. Taken together, our study suggests that mice can use an alternative way of communication between liver and kidney to regulate the daily fluctuations of irRAS components. Identification of this mode of communication could help to better understand the pathophysiology of the kidney, which affects fluid balance and blood pressure.

**Supplementary Information:**

The online version contains supplementary material available at 10.1038/s41598-025-34303-w.

## Introduction

The systemic renin-angiotensin-aldosterone system (sysRAAS) in mammals maintains fluid and electrolyte balance and thus contributes to blood pressure regulation^1^. The sysRAAS originates in the kidney, where renin is secreted in response to a drop in the intrarenal perfusion pressure, low blood Na^+^ concentration, or stimulation by the sympathetic nervous system^2^. Renin converts the liver-secreted angiotensinogen (AGT) to angiotensin I (Ang I) and, subsequently, the lung-derived angiotensin I-converting enzyme (ACE) cleaves Ang I to angiotensin II (Ang II). Binding of Ang II to angiotensin II type 1 receptor (AGTR1) in the adrenal gland releases aldosterone and in blood vessels causes vasoconstriction^3,4^. Aldosterone promotes the kidney to retain Na^+^ and water in the blood^5^. Furthermore, Ang II stimulates the posterior pituitary gland to release vasopressin which increases renal water absorption to regulate blood osmolarity^6^. The combination of vasoconstriction and water/salt retention to increase blood volume normalizes low blood pressure. Finally, Ang II and aldosterone feedback on the release of renin by the kidney to switch off the sysRAAS regulatory cascade^2^. Additional exopeptidases may convert Ang II to Ang III, Ang IV, Ang(1to9) and Ang(1to7), which bind to similar receptors, but have modified and sometimes even opposite activities^7^. Malfunctioning of sysRAAS causes hypertension, while it is possible to treat hypertension with, e.g., ACE or AGTR1 inhibitors^8^. It was described that the activity of sysRAAS and its components followed a circadian rhythm, i.e. showed time-of-day specific differences^9^. Such daily fluctuations may underlie the time-of-day specific increase of heart failures as observed, e.g., in humans^10^.

In addition to the systemic RAAS, there is a local intrarenal renin-angiotensin system (irRAS). The sysRAAS component Ang II is taken up by the proximal tubules of the kidney^11^. Binding of Ang II to AGTR1 in the kidney triggers the production of irRAS components^11–13^. The malfunction of the irRAS disrupts blood pressure regulation and is associated with problems such as cardiac arrest^14–16^. A study revealed that by comparing kidney- and liver-specific *Agt* knock-out mice, only the liver-specific *Agt* knock-out mice showed a significant reduction in intrarenal Ang II, suggesting that hepatic AGT was the primary source of this peptide hormone in the kidney^17^. Thus, problems in blood pressure regulation may arise not only from renal pathologies that disrupt the irRAS, but also from liver disease^1^.

In the liver, many physiological and biochemical functions occur with diurnal rhythms (e.g., the metabolism and plasma protein synthesis). Part of these rhythms is governed by the liver circadian clock^18,19^. These rhythms are driven by the circadian oscillator based on transcriptional–translational feedback loops. In these loops, BMAL1 and CLOCK bind together to form a BMAL1:CLOCK complex, which binds to the E-box regulatory region of the Period (*Per*) genes to activate their transcription. Thus, they act as positive regulators in the loop. Then, the translated PER proteins accumulate and form a complex with the Cryptochrome proteins to repress the transcriptional activity of the BMAL1:CLOCK complex. Thus, they act as negative regulators in the loop^20,21^. A disrupted liver circadian oscillator is implicated in multiple diseases, including metabolic disorder^22^ and cancer^23^. In a recent study, mice with liver-specific *Bmal1* knock-out showed a decrease in systolic blood pressure compared to the control group^24^. The reason is unknown but could be due to a disruption of their sysRAAS and consequently irRAS system^15,16,25^. Interestingly, other investigators reported that there was a close relationship between liver disease and the disruption of the sysRAAS that can lead to several kidney and cardiovascular diseases^1^. Furthermore, patients with liver cirrhosis produced high amounts of angiotensinogen^26^. Interestingly, liver and kidney tissues of mice with colorectal liver metastases both experienced a phase shift in the expression of circadian clock genes^27^, suggesting a signal from the liver metastases acted on both tissues. Hence, the irRAS could be regulated by rhythmic signals from the liver.

The aim of this study was to address whether disruption of the circadian clock in liver affected the irRAS and consequently blood pressure. To this end, we compared normal mice with mice lacking either *Bmal1* or both *Per1* and *Per2* in their hepatocytes. To address changes in the sysRAAS and irRAS, we measured blood pressure, angiotensinogen, renin, ACE and different forms of angiotensin in blood together with expression of sysRAAS components in the liver and lung, and expression of irRAS components. An impact of the circadian clock in the liver on the irRAS would hint towards a dependence of both systems. This finding would help in better understanding the pathophysiology of the kidney, which affects the fluid balance and consequently daily blood pressure.

## Results

### Deletion of hepatic circadian clock genes affects hepatic sysRAAS components

For our study, we compared control mice expressing solely the Cre-recombinase in hepatocytes (LCre) with mice expressing the Cre-recombinase in hepatocytes and floxed *Bmal1* alleles (LBmal1) to inactivate the activating part of the liver circadian oscillator, or mice expressing the Cre-recombinase in hepatocytes and floxed *Period1* and *Period2* alleles (LPer1/2) to inactivate the repressing part of the liver circadian oscillator. Both kinds of mice have consequently a non-functional circadian oscillator in their hepatocytes, while all the circadian oscillators in the other tissues are fully functional.

**Fig. 1:**
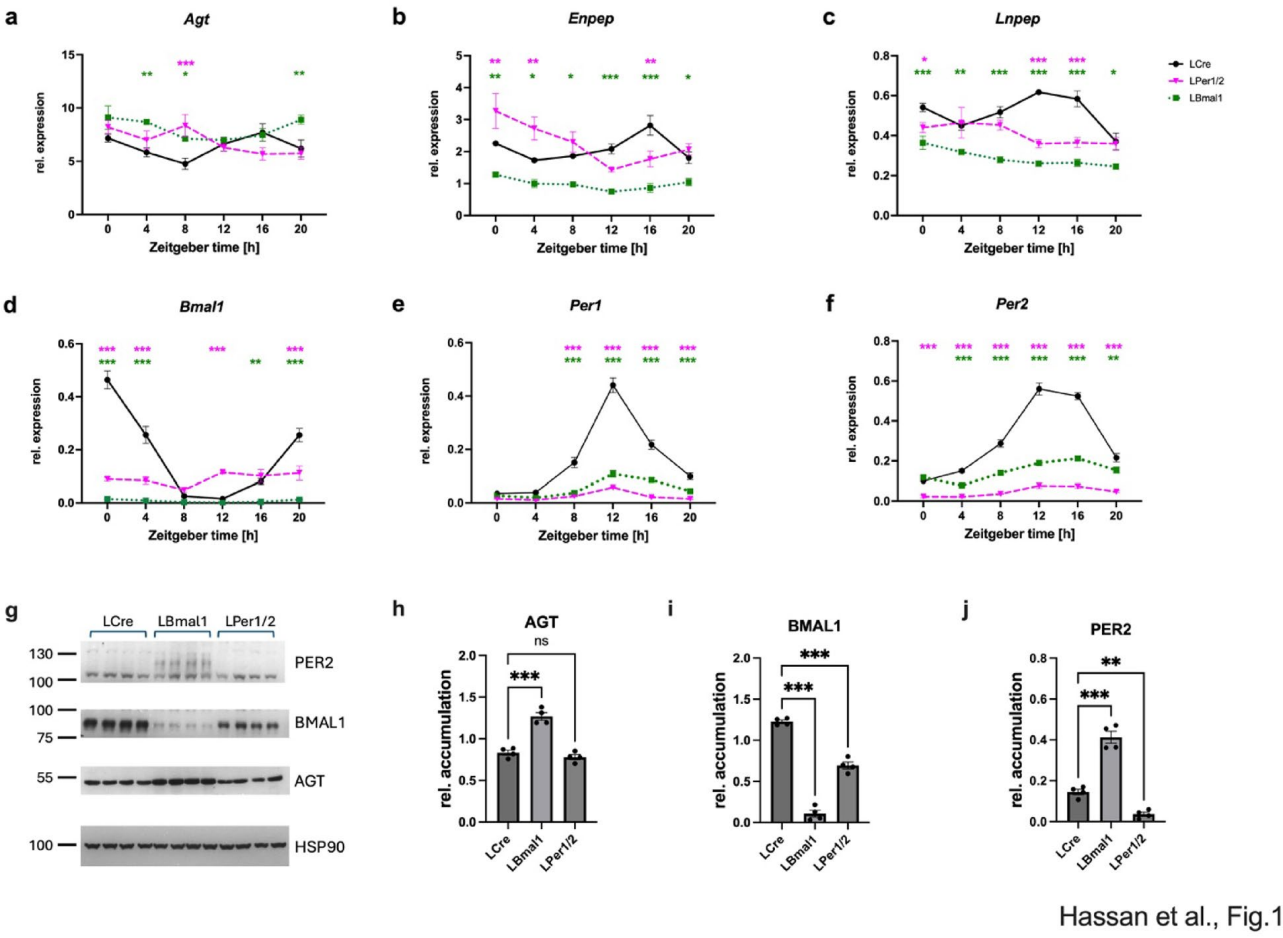
Effect of targeted deletion of hepatic circadian clock genes on liver sysRAAS components. Relative expression of (**a**) *Agt*, (**b**) *Enpep*, (**c**) *Lnpep*, (**d**) *Bmal1*, (**e**) *Per1*, and (**f**) *Per2* in LCre (black solid lines), LBmal1 (green dotted lines) and LPer1/2 (magenta dashed lines) mice at six different *zeitgeber* time points with 4-hour intervals. Green stars indicate significant differences between LCre and LBmal1, and magenta stars indicate significant differences between LCre and LPer1/2. Data are presented as mean ± SEM (n = 3). (**g**) western blot analysis of the indicated proteins at ZT8 and quantification (n = 4) (h-j). On the left are the positions of size markers [kDa]. *Agt*, AGT: Angiotensinogen; *Enpep*: Glutamyl aminopeptidase; *Lnpep*: Leucyl and Cystinyl aminopeptidase; *Bmal1*, BMAL1: Brain and muscle ARNT-like protein 1; *Per1*: Period 1; *Per2*, PER2: Period 2; HSP90: heat shock protein 90. Two-way ANOVA with Tukey’s multiple comparisons test, * p < 0.05; ** p < 0.01; *** p < 0.001.

We first wanted to verify that expression of angiotensinogen (*Agt*) in the liver followed a daily rhythm. Hence, we measured the expression of this gene in the liver of LCre mice over one day. Expression of liver *Agt* was rhythmic (*p* < 0.025, Supplemental table S1l; Fig. 1a). Surprisingly, this rhythmicity remained in LBmal1 (*p* < 0.005) and LPer1/2 (*p* < 0.025) mice albeit with different phases. Compared to LCre (18.43 h), LBmal1 shifted to 23.48 h (*p* < 0.025) and for LPer1/2 to 4.64 h (*p* < 0.0005). The average expression (mesor) of *Agt* in LBmal1 was 26% higher (*p* < 0.0005), while the amplitudes remained the same (*p* > 0.025). The data indicate that liver *Agt* under circadian control peaks in the middle of the dark phase (ZT18.43) but stays rhythmically expressed in absence of the liver circadian clock. Similarly, expression of the exopeptidase *Enpep*, which converts Ang I or Ang II to Ang III, and the receptor for Ang IV, *Lnpep*, in liver-specific knock-out mice was rhythmic and behaved similar to the expression of *Agt* suggesting a coordinated expression of sysRAAS genes in the liver (Fig. 1b, c; Supplemental table S1n, o). As control, we observed reduced expression of *Bmal1* in the liver of LBmal1 mice (*p* < 0.0005, Supplemental table S1m; Fig. 1d), and reduced expression of *Per1* and *Per2* in the liver of LPer1/2 mice (*p* < 0.0005, Supplemental table S1p, q; Fig. 1e, f). The reduction of *Bmal1* in LBmal1 mice reduced expression of *Per1* and *Per2* (*p* < 0.0005; Supplemental table S1p, q), while the reduction of *Per1* and *Per2* in LPer1/2 mice reduced expression of *Bmal1* (*p* < 0.0005, Supplemental table S1m; Fig. 1d). Altogether, both kinds of mice have the expected non-functional circadian oscillator in their hepatocytes. Furthermore, we extracted proteins from liver at ZT8, when the difference in *Agt* expression between the different mouse strain was the most prominent, and probed for AGT, BMAL1 and PER2 (*p* < 0.001, Fig. 1g). AGT accumulation was increased in LBmal1 mice compared to LCre and LPer1/2 (*p* < 0.001, Fig. 1h). BMAL1 was severely reduced in LBmal1 mice and reduced in LPer1/2 mice (Fig. 1i). Finally, PER2 was severely reduced in LPer1/2 and upregulated in LBmal1 (*p* < 0.01 and *p* < 0.001, respectively, Fig. 1j).

### Deletion of hepatic circadian clock genes affects irRAS components in kidney

**Fig. 2:**
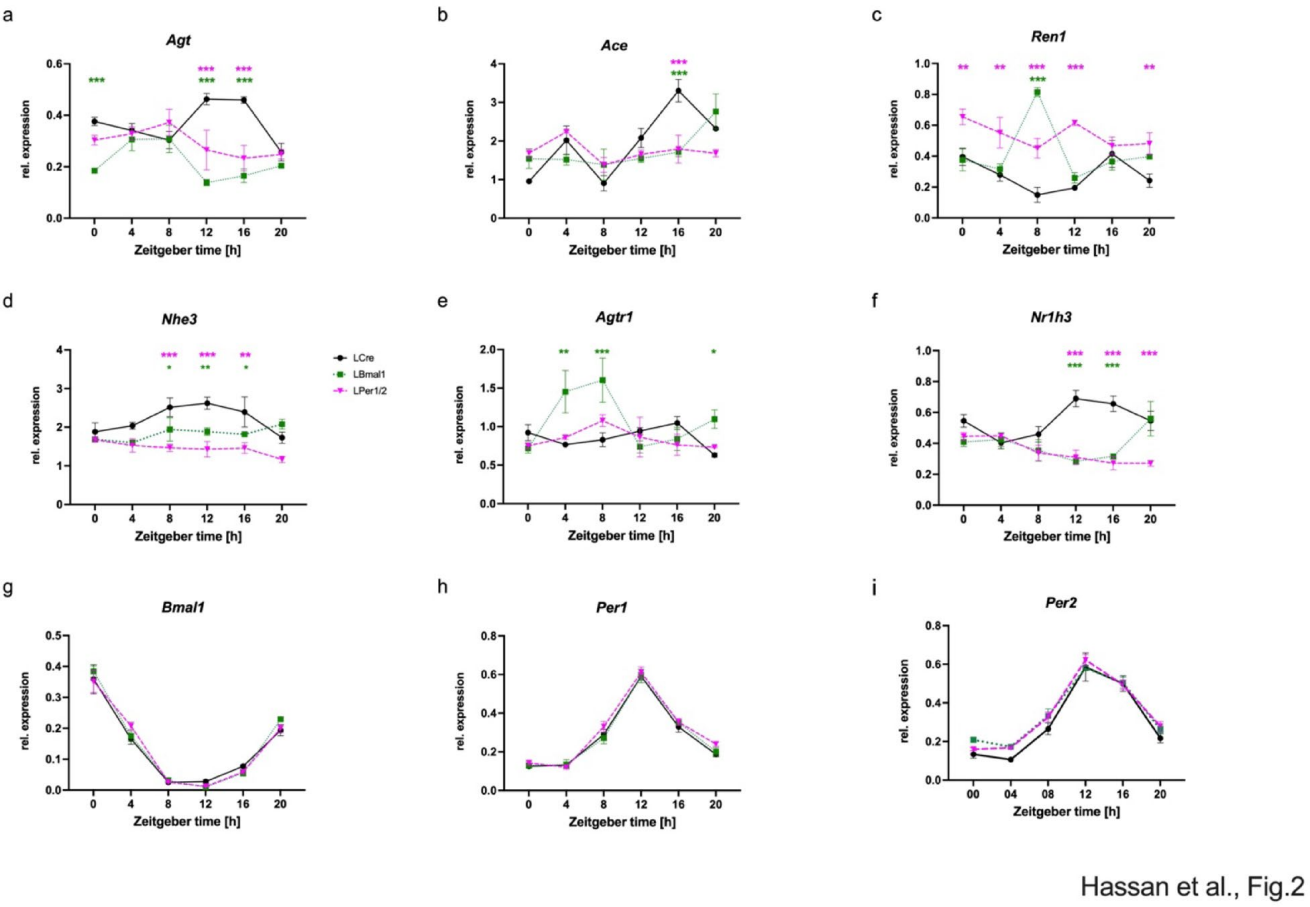
Effect of targeted deletion of hepatic circadian clock genes on irRAS components and renal clock genes. Relative expression of (**a**) *Agt*, (**b**) *Ace*, (**c**) *Ren1*, (**d**) *Nhe3*, (**e**) *Agtr1*, (**f**) *Nr1h3*, (**g**) *Bmal1*, (**h**) *Per1* and (**i**) *Per2* in LCre (black solid lines), LBmal1 (green dotted lines) and LPer1/2 (magenta dashed lines) mice at six different *zeitgeber* time points with 4-hour intervals. Green stars indicate significant differences between LCre and LBmal1, and magenta stars indicate significant differences between LCre and LPer1/2. Data are presented as mean ± SEM (n = 3). *Agt*: Angiotensinogen; *Ace*: Angiotensin I-converting enzyme; *Ren1*: Renin 1; *Nhe3*: Na^+^/H^+^ exchanger 3; *Agtr1*: Angiotensin II receptor type 1; *Nr1h3*: Liver X receptor α; *Bmal1*: Brain and muscle ARNT-like protein 1; *Per1*: Period 1; *Per2*: Period 2. Two-way ANOVA with Tukey’s multiple comparisons test, * p < 0.05; ** p < 0.01; *** p < 0.001.

**Fig. 3:**
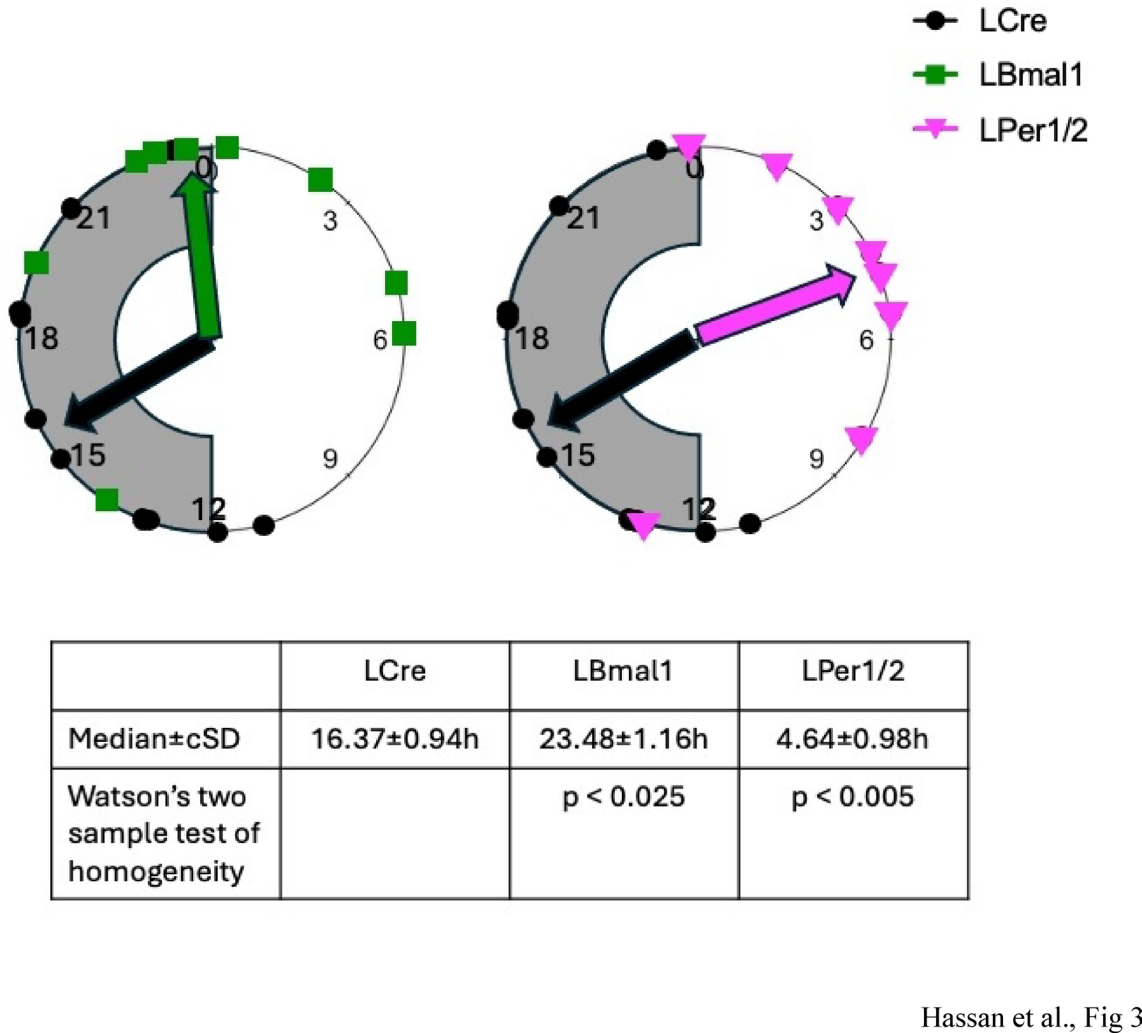
Analysis of the median phase differences for kidney genes in the different genotypes. Left side: Plot of the calculated phases from Supplemental table S1 on a 24 h circle for LCre (black dots) and LBmal1 (green squares) mice. Right side: Plot of the calculated phases from Supplemental table S1 on a 24 h circle for LCre (black dots) and LPer1/2 (magenta triangles) mice. Bottom: calculation of the median phase of gene expression ± circular standard deviation in hours; Watson’s two sample test of homogeneity comparing LCre with LBmal1, or LCre with LPer1/2.

To investigate the possible effect of a targeted deletion of liver clock genes on the irRAS, the expression of some key components was investigated over one day. Renal *Agt* expression was rhythmic in the control group (LCre, *p* < 0.025, Supplemental table S1b; Fig. 2a). Again, in the two hepatocyte-specific knock-out strains, *Agt* expression remained rhythmic (LBmal1, *p* < 0.005; LPer1/2, *p* < 0.025, Supplemental table S1b), while the mesor was significantly reduced (~ 50% for LBmal1, *p* < 0.0005; ~ 25% for LPer1/2, *p* < 0.005). The amplitudes were not affected, but the phases shifted from LCre 13.35 h to LBmal1 4.86 h (*p* < 0.0005) and to LPer1/2 5.51 h (*p* < 0.005). The accumulation of AGT at ZT8 in kidney was slightly reduced in LPer1/2 (Supplemental Fig. S1c, d). Similar diurnal patterns were observed in LCre mice for expression of the irRAS components *Ace* (angiotensin I-converting enzyme, Fig. 2b), *Ren1* (Renin, Fig. 2c), and *Nhe3* (Na^+^/H^+^ exchanger 3, which is important for salt reabsorption, Fig. 2d). The diurnal expression patterns of *Ace*, *Nhe3* and *Ren1* were blunted in LBmal1 and LPer1/2 compared to the control group (Fig. 2b-d; Supplemental table S1a, g,k). The knock-out of *Bmal1* or *Per1/Per2* in the liver differently affected the expression of *Ren1* and *Agtr1* (Angiotensin II receptor type 1, Fig. 2e). *Ren1* was constitutively upregulated in LPer1/2, while *Agtr1* was upregulated in LBmal1 during the light phase (Fig. 2c, e). Finally, expression of the nuclear receptor *Nr1h3/Lxrα*, which may be involved in the regulation of irRAS genes, was rhythmic in all genotypes with reduced mesor and shifted phases in LBmal1 and LPer1/2 mice (Fig. 2f; Supplemental table S1h). The expression of *Enpep* and *Lnpep* in kidney behaved similar to the other irRAS components (Supplementary Fig. S1a, b; Supplemental table S1e, f). Our data hint towards a coordinated regulation of irRAS components in the kidney with different median phases (Fig. 3). The median phase of gene expression shifted from 16.37 ± 0.94 h for LCre to 23.48 ± 1.16 h for LBmal1 (*p* < 0.025) and even further to 4.64 ± 0.98 h for LPer1/2 (*p* < 0.005), which was very close to the estimated phase of liver *Agt* in the three different mouse lines (Supplemental table S1l).

#### Deletion of hepatic circadian clock genes does not affect the renal circadian oscillator

To determine whether these changes in daily patterns of the irRAS components were driven by the circadian molecular clockwork of the kidney, the expression of *Bmal1*, *Per1* and *Per2* was investigated, which function as positive and negative regulators of the circadian molecular clockwork, respectively. The results showed that the diurnal patterns of these circadian genes were similar in the three genotypes (Fig. 2g-i; Supplemental table S1d, i,j) confirming that the liver but not the intrinsic circadian system of the kidney governed the shifts observed in the expression of irRAS components. In line with this, the accumulation of BMAL1 at ZT8 in kidney was similar across all genotypes (Supplemental Fig. S1c, e). The accumulation of PER2 was difficult to judge, because ZT8 represents the minimum of accumulation of this protein during the circadian cycle (Supplemental Fig. S1c, f). Additionally, we investigated the expression of irRAS components in single knock-out mice for *Per1* and *Per2* (LPer1 and LPer2), which do not abolish the function of their liver circadian oscillator, but lack only part of their repressing activity during the circadian cycle. The amplitude of liver *Agt* became blunted in LPer1 and LPer2 mice (Supplemental Fig. S2a). The expression of *Bmal1* in liver was not affected (Supplemental Fig. S2b), while *Per1* was reduced in LPer1 mice (Supplemental Fig. S2c) and *Per2* was reduced in LPer2 mice (Supplemental Fig. S2d). irRAS components were affected in these mice differently to the control group. *Agt* expression in the kidney of LPer1was blunted, but increased in LPer2 (Supplemental Fig. S2e). *Ace* expression was blunted in LPer1 and LPer2 albeit at different expression levels (Supplemental Fig. S2f). *Ren1* expression was also blunted in LPer1 and LPer2 albeit at different expression levels (Supplemental Fig. S2g). The amplitude of *Nhe3* expression was enhanced specifically in LPer1 (Supplemental Fig. S2h). Rhythmic expression of *Nr1h3* was phase-shifted in LPer1 (Supplemental Fig. S2i). Finally, rhythmic expression of *Bmal1*, *Per1* and *Per2* was not affected in the kidney of LPer1 and LPer2 mice (Supplemental Fig. S2j-l). Hence, deletion of *Per1* or *Per2* affected irRAS components accumulation albeit with minor impact on the phase. Taken together, the inactivation of the circadian clock or individual *Per* genes in the liver has strong effects on the irRAS.

### Deletion of hepatic circadian clock genes affects lung *Ace* expression

In the sysRAAS, the lung secretes angiotensin I-converting enzyme (ACE) to convert Ang I to Ang II. Thus, we investigated whether the targeted deletion of liver clock genes can affect *Ace* expression and the intrinsic circadian system of the lung by measuring *Bmal1* and *Per1*. The results showed that the expression of *Ace* in the LCre control group was rhythmic (*p* < 0.005, Supplemental table S1r; Supplemental Fig. S3a). In LBmal1 and LPer1/2 mice, expression of *Ace* lost this rhythmicity (*p* > 0.025 and *p* > 0.025, respectively). For the circadian clock genes in the lung, all three genotypes exhibited time-dependent changes in *Bmal1* and *Per1* expression (Supplemental Fig. S3b, c; Supplemental table S1s, t). While *Bmal1* expression was very similar in the three different mouse strains (Supplemental Fig. S3b), the mesor of *Per1* was higher in LBmal1 and LPer1/2 mice, with LPer1/2 having a peak of *Per1* expression at ZT12 (Supplemental Fig. S3c). Consequently, similar to the kidney, inactivation of the circadian clock in the liver affected *Ace* expression.

### Deletion of hepatic circadian clock affects sysRAAS components in the plasma

**Fig. 4:**
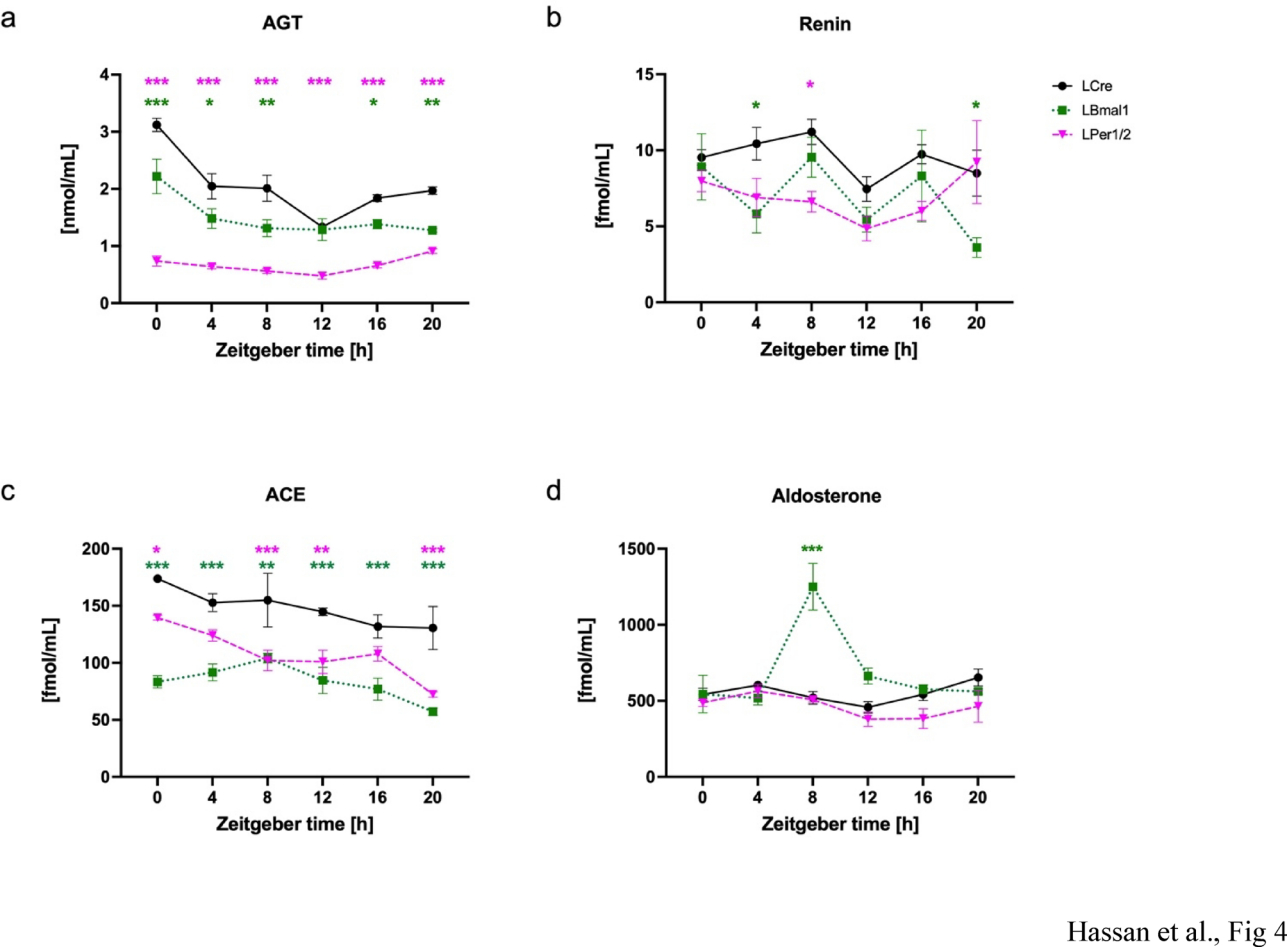
Effect of targeted deletion of hepatic circadian clock genes on plasma sysRAAS components. (**a**) AGT, (**b**) renin, (**c**) ACE, and (**d**) aldosterone in LCre (black solid lines), LBmal1 (green dotted lines) and LPer1/2 (magenta dashed lines) mice at six different *zeitgeber* time points with 4-hour intervals. Green stars indicate significant differences between LCre and LBmal1, and magenta stars indicate significant differences between LCre and LPer1/2. Data are presented as mean ± SEM (*n* = 3). AGT: Angiotensinogen. ACE: Angiotensin I-converting enzyme. Two-way ANOVA with Tukey’s post hoc test, * *p* < 0.05; ** *p* < 0.01; *** *p* < 0.001.

To understand the effects of altered gene expression due to the inactivation of the liver circadian clock on the sysRAAS, the accumulation of the sysRAAS components angiotensinogen (AGT), renin, angiotensin I-converting enzyme (ACE), and aldosterone was measured. In circulating blood, total AGT (Fig. 4a) exhibited a rhythmic pattern in the LCre control group (*p* < 0.0005, Supplemental table S1u) with a peak at 0.43 h. AGT accumulation was still rhythmic in LBmal1 (*p* < 0.025) and LPer1/2 (*p* < 0.0005) but in the two liver-specific knock-out mice the average accumulation was reduced to ~ 50% (*p* < 0.0005 and *p* < 0.0005, respectively). Total renin (Fig. 4b) and ACE (Fig. 4c) levels did not show time-dependent changes in LCre mice (Supplemental table S1v, w). However, both markers had a significant decrease in their levels in LBmal1 and LPer1/2 mice compared to the control group using Two-Way ANOVA (Renin: *p* < 0.01 and ACE: *p* < 0.001, Supplemental table S2v, w; Fig. 4b, c). Aldosterone, the hormone which is secreted from the adrenal gland in response to Ang II, revealed time-dependent changes in all three genotypes (Fig. 4d; Supplemental table S1x). The average accumulation of aldosterone was increased due to a spike at ZT8 in LBmal1 (*P* < 0.025) but overall decreased in LPer1/2 (*p* < 0.005), the amplitude was larger in LBmal1 (*p* < 0.025), and both displayed a change in the maximum of expression compared to LCre (*p* < 0.025). Hence, inactivation of the liver circadian clock impacted the accumulation of sysRAAS components in the plasma.

### Deletion of hepatic circadian clock genes affects blood pressure

**Fig. 5:**
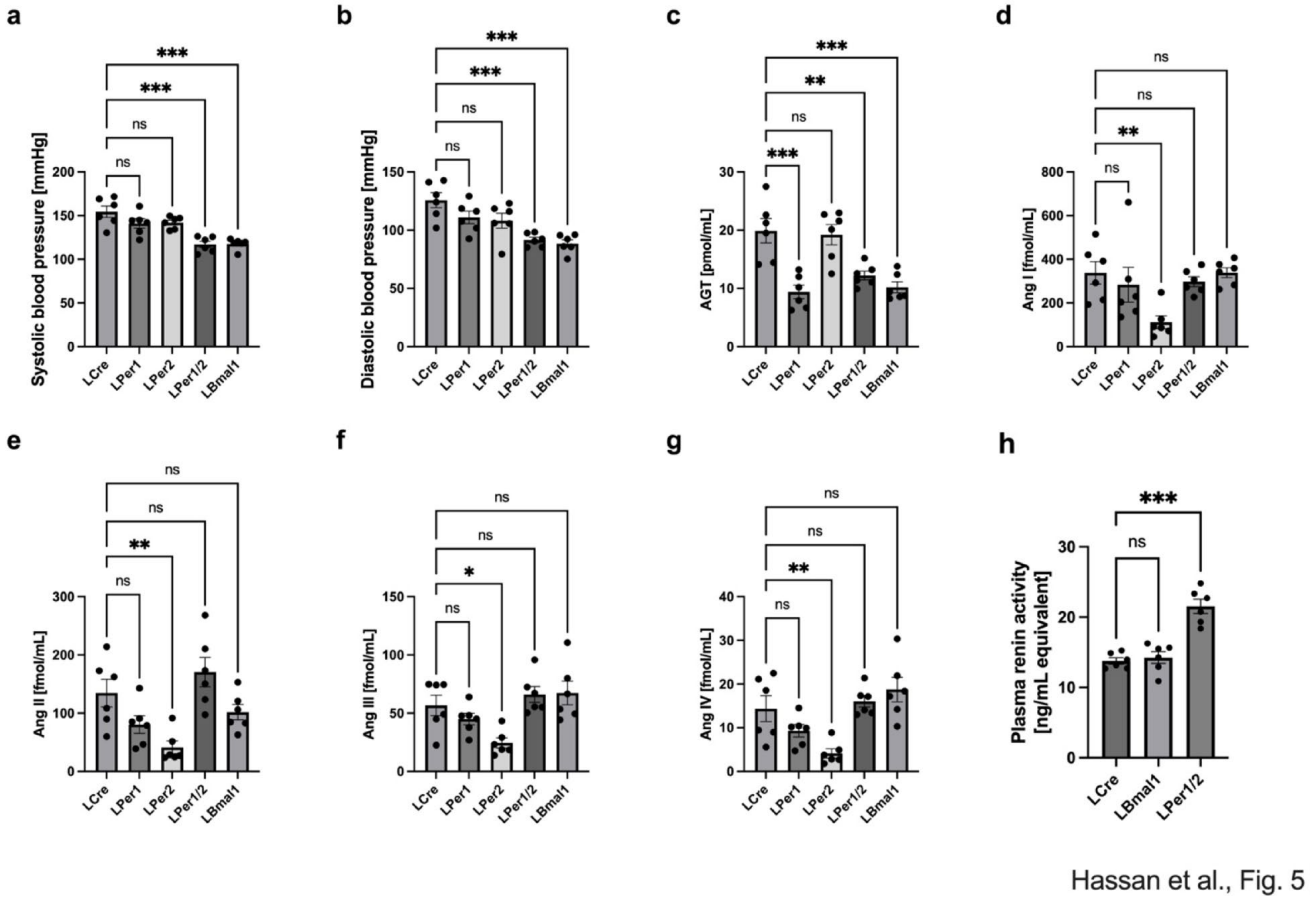
Effect of targeted deletion of hepatic circadian clock genes on blood pressure and peptide hormone accumulation at ZT8. (**a**) systolic and (**b**) diastolic blood pressure of LCre, LBmal1, and LPer1/2 mice. (**c**) concentration of AGT in plasma, (**d**) Ang I, (**e**) Ang II, (**f**) Ang III, (**g**) Ang IV. (**h**) renin activity compared to standard. AGT: angiotensinogen; Ang: angiotensin. Data are presented as mean ± SEM (*n* = 6). One-way ANOVA with Dunnett’s post hoc test compared to LCre, ns: non-significant; * *p* < 0.05; ** *p* < 0.01; *** *p* < 0.001.

Next, we focused on blood pressure and sysRAAS component accumulation in the plasma at ZT8, where we found consistent differences between the hepatocyte-specific knock-out strains and the control mice (Figs. 1, 2, 3 and 4). Both, LBmal1 and LPer1/2 had highly significant reductions (~ 25%) in systolic and diastolic blood pressure (*p* < 0.001 and *p* < 0.001, respectively; Fig. 5a, b). The AGT levels in plasma at this time point were ~ 50% compared to the LCre control mice (*p* < 0.01 for LBmal1 and *p* < 0.05 for LPer1/2; Fig. 5c). Surprisingly, despite reduced AGT levels, the level of Ang I was similar in all three genotypes (Fig. 5d). Ang II, Ang III and Ang IV appeared unaffected in LBmal1 or LPer1/2 compared to the controls (Fig. 5e-g). Finally, LPer1/2 mice had a significantly higher renin activity in their plasma (*p* < 0.001; Fig. 5h), which may compensate for their reduced renin levels at ZT8 (Fig. 4b). By contrast to plasma AGT levels (Fig. 5c), *Agt* expression was elevated in the liver of both LBmal1 and LPer1/2 mice (*p* < 0.001; Supplemental Fig. S4a), while LBmal1 mice had reduced levels of *Enpep* (*p* < 0.001; Supplemental Fig. S4b). The liver genes for subsequent processing of Ang II and III peptide hormones, *Dpp3* and *Anpep*, respectively, and the receptor *Lnpep* were not affected (Supplemental Fig. S4c-e). We also verified that *Bmal1* expression was reduced in LBmal1 mice and *Per1* and *Per2* expression in LPer1/2 mice (Supplemental Fig. S4f-h). Similar to LBmal1 and LPer1/2 mice, LPer1 mice had reduced levels of AGT (*p* < 0.01; Fig. 5c) but only Ang II was reduced among the peptide hormones (*p* < 0.05, Fig. 5d-g). By contrast, LPer2 mice had normal AGT levels (Fig. 5c) while Ang I to Ang IV were highly significantly reduced (Fig. 5d-g). However, despite these changes in peptide hormone levels LPer1 or LPer2 mice had the same systolic and diastolic blood pressure as the LCre control mice (Fig. 5a, b).

## Discussion

Many physiological processes in mammals oscillate in a daily manner^28^. Typical examples include the sleep-wake cycle, body temperature, heart rate, blood pressure, and many more. If these cycles persist under constant conditions, i.e. there is no influence of an external Zeitgeber (time giver), then these processes are most likely governed by the circadian clock. The circadian clock uses a brain structure, the *Suprachiasmatic nuclei* (SCN), to synchronize an organism’s internal rhythms with the external night and day cycle^29^. The SCN relies on neuronal and humoral signals to synchronize downstream circadian clocks in different peripheral organs. For example, coordination of the circadian molecular clockwork in brain with peripheral organs, such as liver and muscles, is very important to keep feeding and activity rhythms^30^ and homeostatic function in muscles^31^. Hence, the circadian timing system is a well evolved system to synchronize the many rhythmic processes within an organism. Ablation of the SCN in rats blunted the diurnal variation in blood pressure^32^. In addition to the coordination between the master clock (SCN) and peripheral organs to regulate different physiological and behavioural parameters, there is crosstalk between peripheral clocks. The liver circadian clock was reported to coordinate the rhythmicity of different peripheral organs (e.g. kidney, lung and heart) in response to feeding regimes regardless their internal clock^33^. Therefore, it is not surprising that a relationship between the circadian clock and the sysRAAS was suggested^9^. Early observations suggested that there were daily changes in renin activity and aldosterone in rats, which shifted after the inversion of the light: dark rhythm^34^. However, later studies in horses showed that these rhythms were affected by the feeding regimen^35^, and lost in patients with ascitic liver cirrhosis or Addison’s disease^36,37^. While the first study was not performed under constant conditions, the latter studies would imply that under disturbance daily rhythms were changed or even lost, which is against the definition of circadian rhythms. Mice without any functional *Bmal1* had severely blunted blood pressure rhythms^38^. Previously, a study was performed with liver-specific knock-out mice of *Bmal1* (i.e. LBmal1)^24^. In this study, the authors observed that these mice had a 10% reduction in systolic blood pressure during the day, suggesting a potential link to the sysRAAS. Since LBmal1 mice have been described as bearing a genetic liver disease affecting their glucose metabolism^39^, we wanted to include at least one more liver-specific knock-out strain without a functional circadian clock in hepatocytes in our study.

Our results showed that in LBmal1 and LPer1/2 mice the rhythmicity of *Agt*, *Enpep*, or *Lnpep* expression in liver was shifted but remained rhythmic (Fig. 1a-c; Supplemental table S1l, n,o). Such residual rhythmcity in tissues without circadian oscillator has been described before. Rhythmic *Per2* expression was found in mice with liver-specific overexpression of NR1D1 ^40^, or in the LBmal1 mice^39^. These rhythmic genes, in absence of a functional circadian clock in the same cell, are probably governed by rhythmic signals entering the tissue from the outside. Such signals could, for example, activate nuclear receptors, which function as co-regulators of circadian clock components^41^. Similarly, such ligands could also override circadian regulation of *Agt* expression to modulate its phase under specific conditions. Since the knock-out of the circadian clock genes was restricted to the hepatocytes, the overall physiology of the animals should be comparable to LCre mice, such as their sleep-wake cycles, body temperature rhythms, feeding rhythms, corticosterone rhythms, or the cyclic function of their sympathetic nervous system. However, we can not rule out the possibility that a signal from the liver reaches the brain, in particular the SCN, and then signals to all the other organs to synchronize the sysRAAS. Such a potential signal had then to be 7 h phase-shifted in LBmal1 mice or phase-inverted (12 h shifted) in LPer1/2 (Fig. 3).

Interestingly, we found the same scenario to occur in the kidney but not the lung (Fig. 2a-f, Supplemental Fig. S3a). For both tissues, it is tempting to speculate that a direct or indirect signal from the liver was missing to express the irRAS components in their original phase (kidney) or to maintain the rhythmicity of *Ace* (lung). In line with our observation, it was shown that signals from the liver affected rhythmic gene expression in perivascular adipose tissue and changed the energy metabolism^24^. Unfortunately, we do not know yet which signal from the liver is used to synchronize the irRAS. From our data, angiotensinogen, or the AGT-derived peptide hormones, Ang I to Ang IV, in the plasma do not appear to be appropriate candidates, because in LPer1 AGT is as low as in LBmal1 or LPer1/2, but there is no impact on the blood pressure (Fig. 5). Similarly, the peptide hormones Ang I to Ang IV were reduced in LPer2, again without impact on the blood pressure (Fig. 5). People have suggested that not the sysRAAS components in plasma, but the irRAS components in the kidney were a better predictor of blood pressure^14,42^. Indeed, the reduced levels of renin and ACE found in the plasma (Fig. 4b, c), which would explain a reduced blood pressure, do not correlate well with the amount of processed Ang I and Ang II in the plasma (Fig. 5d, e). The aldosterone levels in LPer1/2 were similar to LCre, while we do not have an explanation for the surge of aldosterone observed in LBmal1 mice (Fig. 4d) since there were even reduced levels of Ang II (Fig. 5e), which by binding to AGTR1 in the adrenal gland releases aldosterone^3^. The difference in aldosterone levels between LBmal1 and LPer1/2 would therefore not explain the reduced blood pressure of both mouse strains measured at the same time. Oxysterols, which come from the hepatic cholesterol metabolism and function as ligands for the nuclear receptor NR1H3/LXRα, are secreted by the liver and affect the sysRAAS system located in the brain^43^. However, such a role for oxysterols and NR1H3 to coordinate the irRAS in the kidney has to be investigated in the future.

It is well known that liver is the major metabolic platform in the body, and all systemic metabolic processes are under its control. Most of these metabolic processes exhibit circadian rhythms which are regulated by the liver circadian molecular clockwork^44,45^. Disruption of the liver clock genes was reported to be associated with imbalance in systemic metabolism and metabolic diseases^46^. This metabolic imbalance was shown to be related to changes in the irRAS as well as dysregulation of blood pressure^47–49^. Also, it was reported that metabolic imbalance increases inflammation and oxidative stress which subsequently affects the irRAS components^50^. All these factors could be another explanation for the changes in the irRAS components and blood pressure which were observed in our study.

The components of the sysRAAS as measured in the plasma were not compatible with the observed blood pressure phenotype. Hence, we were looking for component(s) of the irRAS that could plausibly explain the reduced blood pressure observed in LBmal1 and LPer1/2 mice, and the absence of such a phenotype in LPer1 and LPer2. There were two genes whose expression was blunted in the kidney of LBmal1 and LPer1/2, while unaffected in LPer2 and increased in LPer1. Furthermore, the expression of these genes in LCre followed a diurnal rhythm with a peak during the activity phase of a mouse, when there is an increase in its blood pressure^38^. The first gene was the Na^+^/H^+^ exchanger 3 (Fig. 2d). NHE3 facilitates the exchange of sodium ions for hydrogen ions to regulate sodium and water balance^51^. There are no human diseases known involving *Nhe3*, but kidney-specific as well as renal tubule-specific knock-out mice of this gene have a low blood pressure consistent with our findings^52–54^. It was described that *Nhe3* was regulated by BMAL1 in mice and rats^55^. This observation was not compatible with our data because rhythmic Nhe3 expression was blunted in LBmal1 and LPer1/2 mice, which have a normal circadian oscillator in their kidney (Fig. 2d, g-i). It is tempting to speculate that NHE3 and probably further, not yet investigated blood pressure-regulating factors in the kidney are plausible contributing factors for the observed phenotype. The second gene was the nuclear receptor *Nr1h3*/*Lxrα* (Fig. 2f). Its expression profile was very similar to *Nhe3* in all the different mouse strains investigated. In contrast to *Nhe3*, much less is known about the function and regulation of rhythmic expression of this gene in the context of the irRAS.

**Fig. 6:**
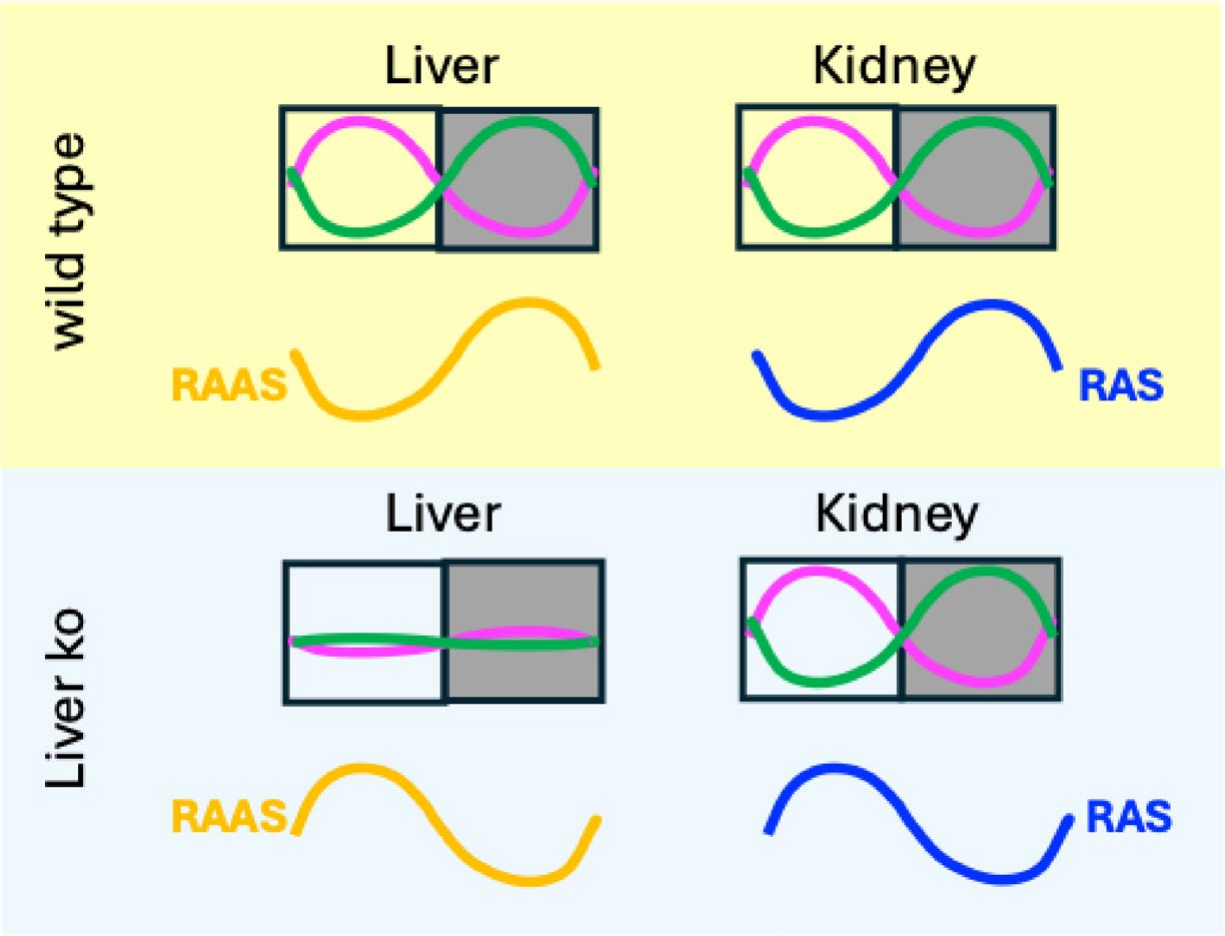
Model. Comparison of two different tissues in two different mouse strains. In wild type mice (yellow box), liver and kidney have a functional circadian oscillator: *Per1* and *Per2* expression peak in the light phase (magenta), while *Bmal1* expression peaks in the dark phase (green). The systemic RAAS (orange) and intrarenal RAS (blue) components are synchronized and peak during the dark phase. In liver-specific knock-out mice (light blue box) there is no functional oscillator in the liver, while the circadian oscillator in the kidney remains unaffected. Surprisingly, the systemic RAAS and the intrarenal RAS components are still partially synchronized albeit their peak is shifted towards the light phase.

Taken together, we found that the irRAS was affected by yet unknown signals from the liver. An impact of the circadian clock in the liver to synchronize gene expression in the kidney is less likely (Fig. 6). Our results also suggested one irRAS component, NHE3, to be involved in the rhythmic regulation of blood pressure by the kidney.

### Limitations

To completely rule out a function of the circadian oscillator in the kidney to synchronize the irRAS, a tissue-specific knock-out mouse strain had to be created. However, by contrast to the normal liver, where only hepatocytes secrete AGT, the kidney is much more heterogenous. Hence, a single knock-out mouse would probably not be sufficient to reach a conclusion. Similarly, we can not rule out that the observed phenotype of down-regulation of *Nhe3* was dominant over the action of the circadian oscillator. However, this possibility seems less likely, because the phase relationships of many genes changed at the same time in liver and kidney (Figs. 1 and 2). Furthermore, our study is not in agreement with studies using a total knock-out of *Bmal1* ^38^, or in rats with ablated SCN^32^. Such animals have probably other physiological problems, e.g., in body temperature and feeding cycles, or in the activity of their sympathetic nervous system. Therefore, it is difficult to compare those studies with ours. Finally, there is a conflict between the RNA accumulation of *Agt* in the liver (higher in LBmal1 and LPer1/2, Fig. 1A, Fig. S4A) and AGT found in the plasma (lower in LBmal1 and LPer1/2, Figs. 4A and 5C). The reason for this discrepancy is not known yet, but may involve either a blocked secretion from the liver, a reduced half-life, or higher clearing rate of AGT in the plasma. As conclusion, further experiments are necessary to identify the rhythmic signal from the liver and understand how this synchronizing signal affects the pathophysiology of the kidney and consequently its impact on blood pressure regulation.

## Materials and methods

### Selected mouse strains

Blood pressure is known as a sexually dimorphic feature because it is influenced by sex hormones (e.g. testosterone in males and estrogen in females)^56^. In female mice the blood pressure is highly affected by the estrous cycle^57^. Thus, to get solid and consistent data, only male mice were used. We used five different mouse strains, which all express one copy of Cre-recombinase under the control of the hepatocyte-specific albumin promoter. Liver-Cre mice (LCre; RRID: IMSR_EM:00603) were used as a control group, in addition to 4 mouse strains with floxed (fl.) alleles to delete circadian clock genes in hepatocytes: LCre x *Bmal1*fl/fl (LBmal1; RRID: IMSR_JAX:007668), LCre x *Per1*fl/fl (LPer1; RRID: IMSR_EM:14846), LCre x *Per2*fl/fl (LPer2; RRID: IMSR_EM:10599) and LCre x *Per1*fl/fl/*Per2*fl/fl (LPer1/2; RRID: IMSR_ EM:14846 x RRID: IMSR_EM:10599). Both LBmal1 and LPer1/2 mouse strains have non-functional circadian oscillators and therefore abolished circadian rhythms in hepatocytes, while LPer1 and LPer2 are only partially inhibited. Apart from the hepatocytes, the circadian clock is functional in all other cell types and tissues. Reporting on animal experiments was performed according to the ARRIVE guidelines. All mouse strains were bred and genotyped in our animal facility. If possible, we used animals originating from different breeding pairs. At the age of 3–5 months, randomly chosen male mice were placed in an isolated cabinet with light/dark schedule (12 h:12 h) and stable temperature and humidity. Animals were adapted to these conditions for 7 days before performing the experiment. A cardboard tube was added to each cage to accustom the animals to the blood pressure experimental conditions. Food and water were freely available (i.e. *ad libitum*). All experiments and procedures were performed according to the Schweizer Tierschutzgesetz guidelines and approved by the Canton of Fribourg and their commission for animal experiments (2024-33-FR, 37384).

### Collection of blood plasma and tissues

Organs (liver, kidney and lung) and blood plasma were collected at different Zeitgeber time points (ZT) (ZT0 indicating light on and ZT12 indicating light off) in 4 h intervals according to the following schedule (ZT0, ZT4, ZT8, ZT12, ZT16 and ZT20). Collection during the dark phase was performed under dim-red light. To collect blood plasma and tissue samples from different mice over one day, animals (18 mice from each genotype; 3 mice/ ZT) were euthanized by intraperitoneal injection of a ketamine (Narketan 10, Vetoquinol AG, Bern, Switzerland) and medetomidine (Dormitor, Orion Pharma corporation, Espoo, Finland) mixture (240 mg/kg ketamine and 0.9 mg/kg medetomidine) in sterile 0.9% NaCl. Blood samples were collected by cardiac puncture in lithium heparin blood tubes (LH, Sarstedt AG & CO KG, Nümbrecht, Germany) on ice and quickly mixed to avoid coagulation. The blood was then centrifuged at 2,000 x g for 15 min at 4 °C, and the separated plasma was transferred to low-protein binding tubes and stored at -80 °C until analysis. After blood collection, organs to be analyzed by real-time PCR (RT-PCR) were excised, snap frozen in liquid nitrogen and stored in -80 °C until being extracted. For the analysis at ZT8, 4 mice per genotype were used.

### Analysis of mRNA expression by real-time PCR

Total RNA from kidney and lung was extracted with the NucleoSpin RNA extraction kit (Machery-Nagel, Düren, Germany) following the manufacturer’s instructions. Total RNA from liver was extracted with 600 µL of TriPure isolation reagent (Roche diagnostic GmbH, Mannheim, Germany). Briefly, after homogenizing the sample, the samples were centrifuged, and the supernatants were transferred to new tubes and mixed with 60 µL of 1-bromo-3-chloropropane (Sigma Aldrich, St. Louis, USA). The samples were kept on ice for 5 min and then centrifuged for 5 min at 12,000 x g at 4 °C. Then, the clear phases from the samples were transferred to new tubes and mixed with an equal amount of 70% ethanol. This mixture was loaded on RNA purification column from the EXTRACTME total RNA kit (Blirt, Gdansk, Poland) and the manufacturer’s instructions were followed. The total concentrations of RNA and the purity of samples were measured using a Nanodrop1000 (Thermo Scientific, USA). 1 µg of RNA was converted to cDNA using SuperScript IV VILO Master mix (Thermo Fisher Scientific, Vilnius, Lithuania) following the manufacturer’s instructions. Each cDNA sample was diluted with 200 µL RNAse free water. For 15 µL reaction volume of RT-PCR, 5 µL of diluted cDNA was mixed with 2.5 µL of 5.4 mM forward and reverse primers and 1.2 mM TaqMan probe (Supplemental table S3) and 7.5 µL of KAPA probe fast universal RT-PCR master mix (Merck, Darmstadt, Germany). The RT-PCR of different genes was performed using a Corbett Rotorgene RG-6000 machine with a Rotor-disc 100 rotor and Rotor-Gene Q series software (version 2.3.4) (QIAGEN, Hilden, Germany). For all probes, we used a threshold of 0.016. The genes measured in liver and kidney over the day were normalized according to the expression of *Nono*, in lung against *Gapdh*, and the liver genes at ZT8 were normalized to the geometric mean of *Nono*, *Sirt2*, *Cdk5*, *Wdr5* and *Atp5h*.

### ELISA measurements

Plasma proteins and aldosterone were measured using enzyme-linked immunosorbent assay (ELISA) kits (Mouse ACE ELISA Kit, # ELM-ACE, RayBiotech, Peachtree Corners, GA, USA; mouse Renin 1 ELISA Kit, # ELM-Renin1, RayBiotech, Peachtree Corners, GA, USA; Mouse AGT ELISA Kit, #JOTEK0636Mo, Jotbody, Hong Kong, China; and aldosterone ELISA Kit, #JOTEK0561Mo, Jotbody, Hong Kong, China) according to the manufacturer’s instructions. Samples were diluted 1:500 for ACE and 1:50 for renin in assay diluent A buffer provided by their kit. Concentrations were calculated according to the co-measured standard curve and then multiplied by the dilution factor (for ACE and renin only).

### Renin activity

The renin activity in 50 µL of plasma was measured in 10 min intervals over 2 hours as described^58^ and compared to a purified renin standard of the kit used (MAK157, Merck, Germany). The time-point measurements for each sample were constant over the 2 hours (linear regression, R^2^ > 0.95).

### Quantification of angiotensin peptides

To quantify angiotensin peptide hormones, 150 µL plasma was complemented with heavy isotope-labelled Ang I and Ang II, and Ang III and Ang IV (13C6-15N1 isoleucine modification; GenScript Biotech, Nanjing, China) to a final concentration of 2.5 nM and 0.25 nM, respectively. The plasma proteins were denatured with 8 M urea final concentration, before being precipitated with an equal volume of acetonitrile. The samples were then centrifuged at 10,000 x g for 10 min. The supernatant containing the peptide hormones was lyophilized to dryness, resuspended in 400 µL water and the pH adjusted to < 2 with trifluoroacetic acid. To remove unwanted proteins and salts, the sample was passed through a 10 kDa cut off filter (Vivacon 500, Sartorius) and desalted using stage tips^59^. Peptides were then separated on a ReproSil-Pur 120 C18-AQ column (1.9 μm beads, Dr. Maisch, Tübingen, Germany) using a gradient from 0 to 80% acetonitrile in 0.1% formic acid and directly applied to an Orbitrap Exploris 480 mass spectrometer (Thermo Fisher Scientific, Waltham, MA, USA). The conditions were: spray voltage (2.3 kV), ion-transfer tube temperature (250 °C; no sheath and auxiliary gas were used), parallel reaction monitoring (PRM) mode (at 30,000 resolution), AGC target (set to 5 × 10^5^), isolation window (set to 1.6 m/z) and maximum injection time of 54 milliseconds. Data were acquired in centroid mode and were analyzed with Skyline (MacCossLab, University of Washington) using MS/MS spectra from the synthetic peptides.

### Blood pressure measurements

Systolic and diastolic blood pressure were measured at ZT8 in different genotypes using a non-invasive mouse tail-cuff system (CODA 3, software 4.2, Kent Scientific, Torrington, USA) following the manufacturer’s instructions and as described in ^60^. Animals were labeled A1 to A6 and the genotype unknown to the experimenter. For each genotype, the experiments lasted 8 days. The first 5 days were an adaptation period for the handling and experimental procedure to reduce stress and handling-related effects. During the adaptation sessions, animals were subjected to a gradual increase in measuring cycles till they reached a total of 30 cycles of inflation and deflation on day 5 to measure the blood pressure in their tail. All data from the first 5 days were excluded from the calculations. The animals were then subjected to 3 experimental days with 30 cycles of inflation and deflation, and the average of the three days was calculated. No data were excluded from the analysis.

### Western blot analysis

Pieces from liver or kidney (~ 100 mg tissue) were homogenized in 200 µL 20% glycerol, 100 mM KCl, 20 mM Hepes pH 7.6, 0.2 mM EDTA, 2 mM DTT and 1 x protease inhibitor (Complete Ultra tablets, Roche, Mannheim) using a hand-held homogenizer with plastic pestle. Using a vortex machine, 200 µL of a solution of 50 mM Hepes pH 7.6, 600 mM NaCl, 2 M urea, 2% Nonident NP-40 and 1 x protease inhibitor (Complete Ultra tablets, Roche, Mannheim) was slowly added. After an incubation of 30 min on ice, the samples were centrifuged at 17,000 g, 4 °C. The supernatant was then mixed 3:1 with 4x Laemmli buffer and boiled for 5 min. Equal amounts of protein were loaded on 7.5% SDS-PAGE to detect BMAL1 and PER2, or 10% SDS-PAGE to detect AGT and HSP90. Antibodies for BMAL1 and PER2 have been described^41^, while we used anti-HSP90a/b (sc-13119, Santa Cruz, Dallas, TX, USA) and anti-AGT (79299 S, Cell Signaling Technology, Danvers, MA, USA) and the appropriate secondary antibodies coupled to horseradish peroxidase. Antibody/protein complexes were visualized using ECL (RPN2232, Cytiva, Marlborough, MA, USA) and exposure to film (CL-X Posure Film, Thermo Fisher Scientific, Waltham, MA, USA). The films were scanned with a GelDoc XR + system (Bio-Rad laboratories, Berkeley, CA, USA) and analyzed with the ImageLab software (6.0.1 build 34). Protein accumulation in liver and kidney was normalized to HSP90.

### Statistical analysis

CircaCompare was used to determine the rhythmicity of relative gene expression and the accumulation of proteins and hormones in blood^61^. Because of comparing LCre with LBmal1 and LPer1/2, i.e. multiple testing, we used Bonferroni-corrected p-values to judge for rhythmicity. If rhythmic (*p* < 0.025), the mesor, amplitude and phase of the mouse strains were compared to LCre mice (Supplemental table S1). The phases were further analyzed using the R-package Circular (version 0.5-1; run under R version 4.2.2) ^62^ to calculate a theoretical median phase ± the circular standard deviation of the kidney genes (not considering *Bmal1*, *Per1* and *Per2*) in a given genotype. Watson’s two sample test of homogeneity was then used to determine if the distributions were identical or not. Further statistical analysis was performed using the GraphPad Prism software (GraphPad Software, Boston, MA, USA; version 10.3.1) (Supplemental table S2). Two-Way ANOVA followed by Tukey’s multiple comparisons test was used to determine the differences between groups according to time points and genotypes. One-Way ANOVA followed by *Dunnett’s* multiple comparisons test was used to compare more than two groups at ZT8 for gene expression, blood pressure and mass spectrometry parameters.

## Supplementary Information

Below is the link to the electronic supplementary material.

Supplementary Material 1

Supplementary Material 2

Supplementary Material 3

Supplementary Material 4

Supplementary Material 5

Supplementary Material 6

Supplementary Material 7

## Data Availability

All data are available in the main text or the supplementary materials.
