# Supplementary figures and images for: Loss of the liver circadian clock affects the expression of intrarenal renin-angiotensin system components
Source: Sci Rep. 2025 Dec 29;16:4158. doi: 10.1038/s41598-025-34303-w (PMC12859090; doi:10.1038/s41598-025-34303-w)

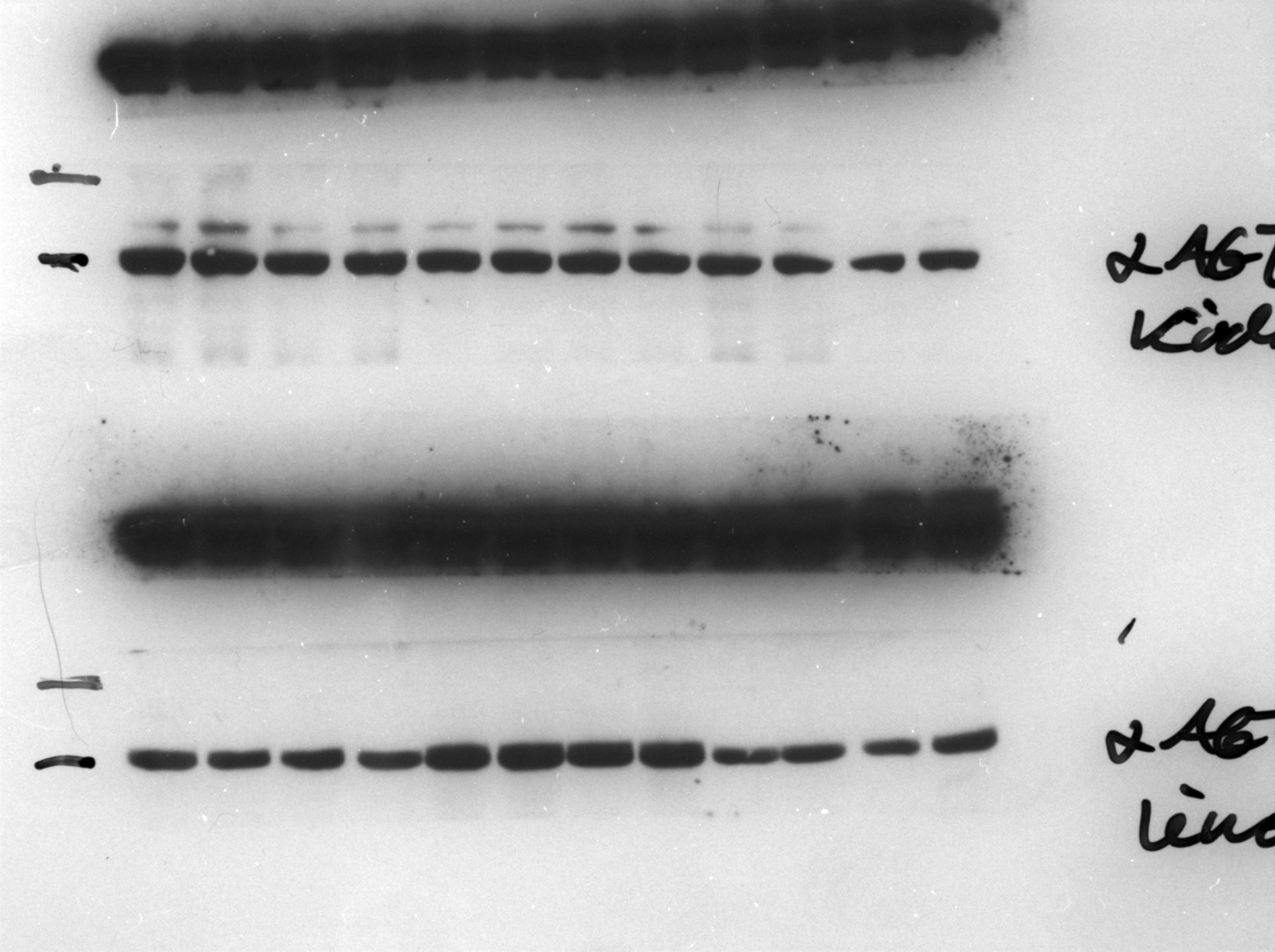

Supplement: Supplementary file 1 — Supplementary Material 1 [file 41598_2025_34303_MOESM1_ESM.jpg]

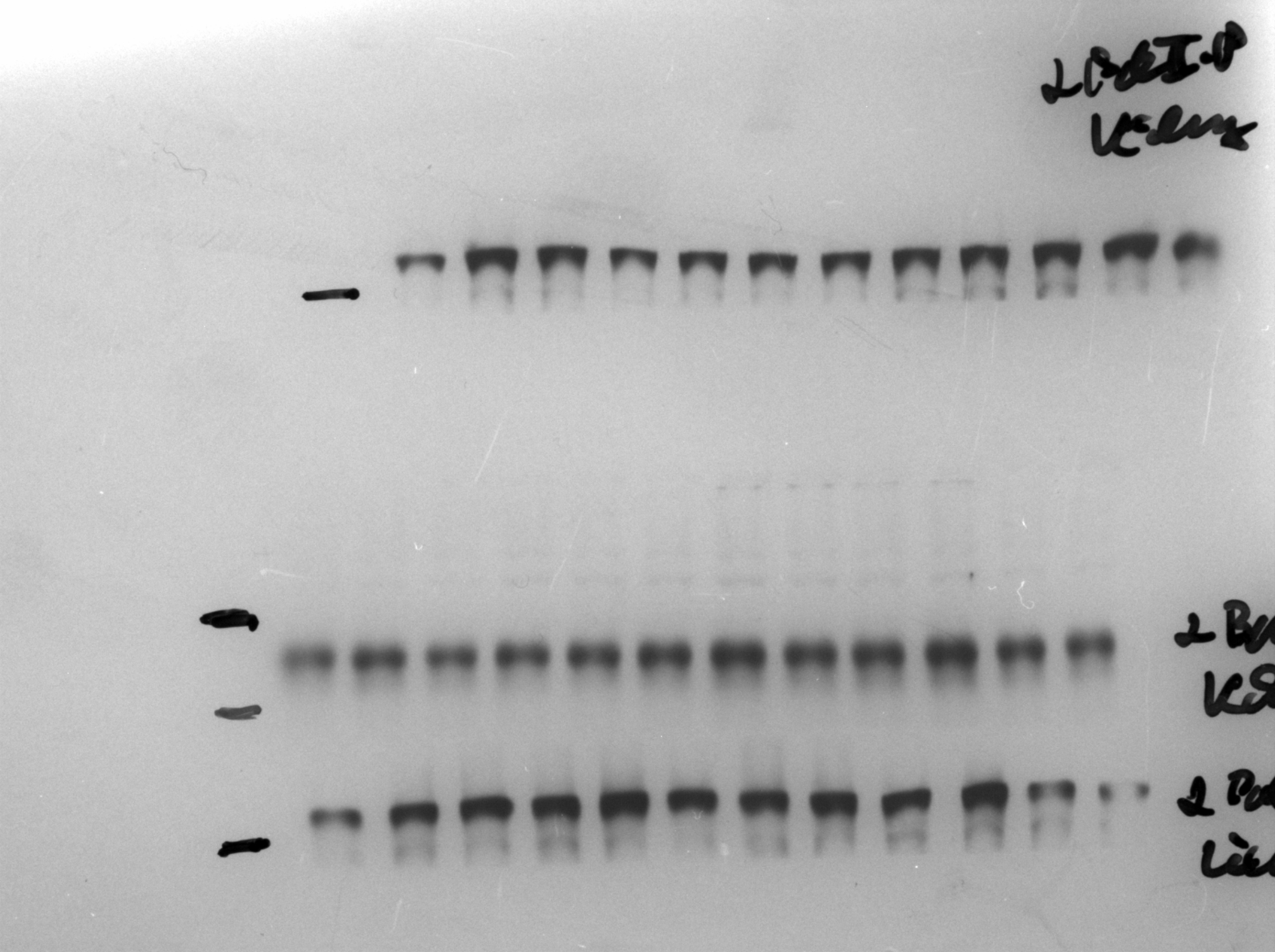

Supplement: Supplementary file 2 — Supplementary Material 2 [file 41598_2025_34303_MOESM2_ESM.jpg]

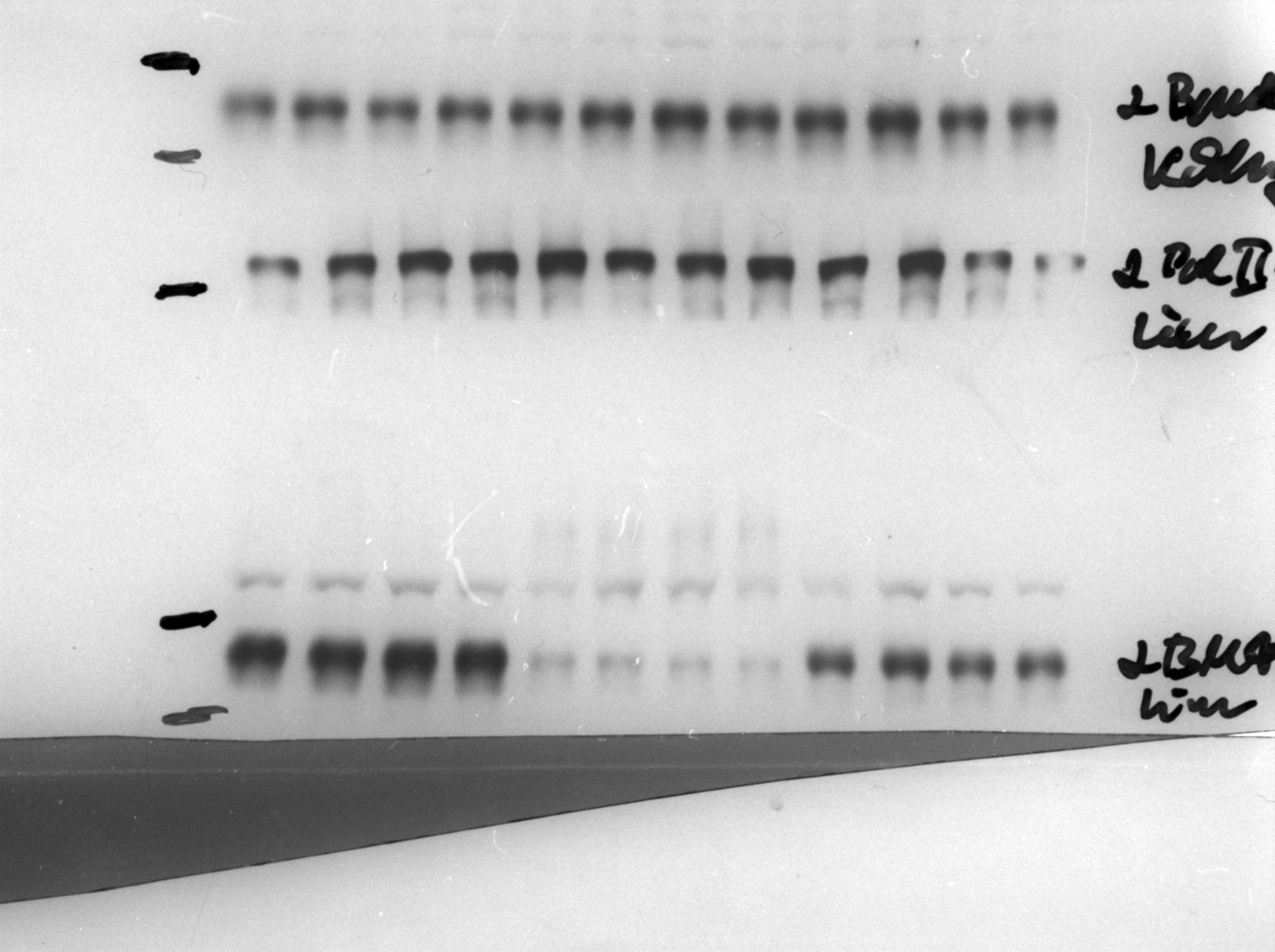

Supplement: Supplementary file 3 — Supplementary Material 3 [file 41598_2025_34303_MOESM3_ESM.jpg]

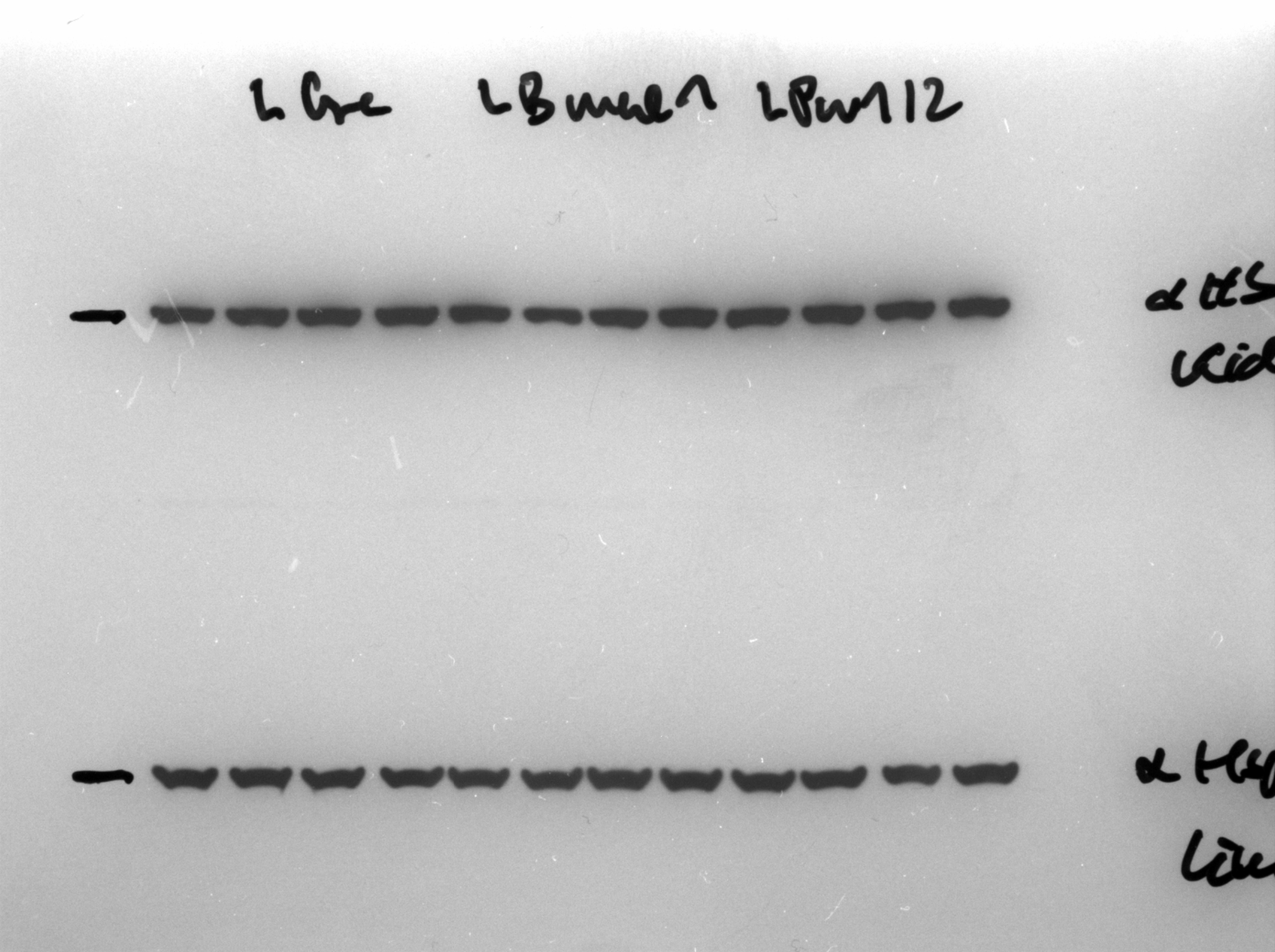

Supplement: Supplementary file 4 — Supplementary Material 4 [file 41598_2025_34303_MOESM4_ESM.jpg]

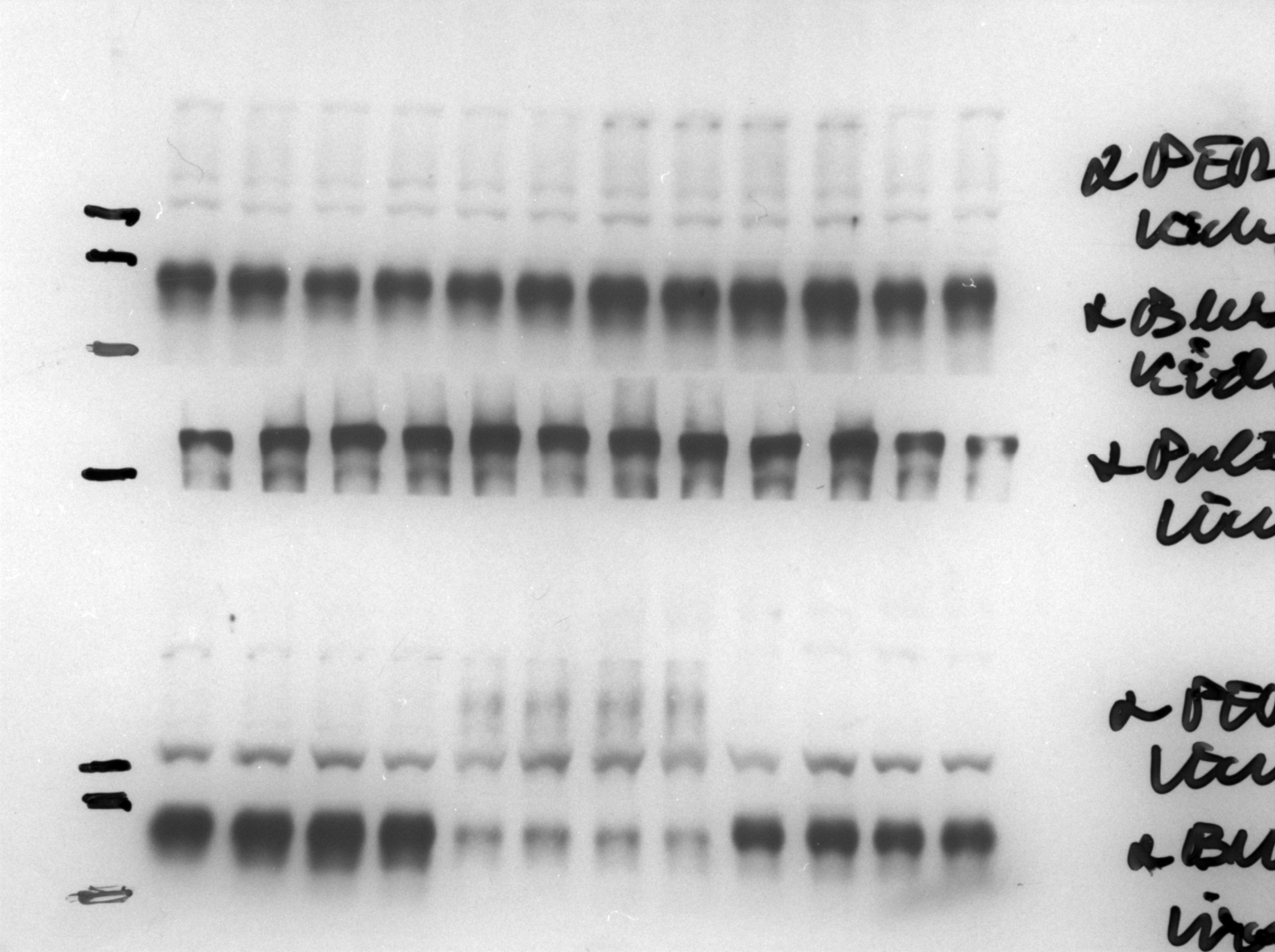

Supplement: Supplementary file 5 — Supplementary Material 5 [file 41598_2025_34303_MOESM5_ESM.jpg]
