# Supplementary material for: Loss of the liver circadian clock affects the expression of intrarenal renin-angiotensin system components
Source: Sci Rep. 2025 Dec 29;16:4158. doi: 10.1038/s41598-025-34303-w (PMC12859090; doi:10.1038/s41598-025-34303-w)
Supplement: Supplementary file 6 — Supplementary Material 6 [file 41598_2025_34303_MOESM6_ESM.pdf]

Supplemental table S1. List of the statistical parameters and significance values for each gene and plasma RAAS components. The table depicts the output parameters from the program CircaCompare [61]. For each genotype and gene measured, there is a p value describing the probability for rhythmicity of the fitted curve, the mesor, the amplitude, and the peak of gene expression. Note that the program fits cosinusoidal curves to the gene expression profiles and from those determines the parameters, but **not** from the curves displayed in the figures. Red color indicates that the mesor, amplitude, or peak is not significantly different compared to LCre, or the gene is not rhythmic. *Ace*: Angiotensin I-converting enzyme; *Agt*: Angiotensinogen; *Agtr1*: Angiotensin II receptor type 1; *Anpep*: Alanyl aminopeptidase; *Bmal1*: Brain and muscle ARNT-like protein 1; *Dpp3*: Dipeptidyl peptidase; *Enpep*: Glutamyl aminopeptidase; *Lnpep*: Leucyl and Cystinyl aminopeptidase; *Nhe3*: Na^+/H^+ exchanger 3; *Nr1h3*: Liver X receptor α; *Per1*: Period 1; *Per2*: Period 2; *Ren1*: Renin 1; *Rnpep*: *Arginyl aminopeptidase* . p for rhythmicity: $p < 0.025$; $p < 0.005$; $p < 0.0005$ (Bonferroni-adjusted p-values for multiple testing); p for mesor, amplitude and phase: $p < 0.025$; $p < 0.005$; $p < 0.0005$ (Bonferroni-adjusted p-values for multiple testing).

	p for rhythmicity	Mesor	Amplitude	Peak
**Kidney**				
a)Ace				
LCre	0.00256926	1.9308208	0.855614	16.38
LBmal1	0.017060765	1.7451201	0.493219	19.55
p-value		0.3882422	0.236423	0.104694
LPer1/2	0.282627135	1.7386149	0.142559	1.18
p-value		0.3208665	0.012787	0.099123
b) Agt				
LCre	0.018515621	0.3632573	0.065115	14.75
LBmal1	0.004017816	0.283103	0.098869	5.30
p-value		9.45E-07	0.69061	0.00018
LPer1/2	0.019036297	0.2432741	0.063065	5.33
p-value		0.006211	0.003785	0.001345
c) Agtr1a				
LCre	0.127853556	0.8563481	0.089989	12.99
LBmal1	0.029512323	1.0747301	0.323485	5.87
p-value		0.0423026	0.119295	0.126852
LPer1/2	0.04236756	0.8411124	0.149136	8.10
p-value		0.8069211	0.503682	0.120956

d) Bmal1

LCre	9.12E-08	0.1438226	0.158771	23.17
LBmal1	3.25E-07	0.1259761	0.157668	22.49
p-value		0.314477	0.964638	0.261313
LPer1/2	3.32E-09	0.105842	0.12531	23.78
p-value		0.0099823	0.096921	0.260437

e) Enpep

LCre	0.040104204	2.4132069	0.583083	18.53
LBmal1	0.103686463	1.8247514	0.277146	18.24
p-value		0.0104928	0.323568	0.930503
LPer1/2	0.257566275	1.6588092	0.075685	5.59
p-value		0.0003914	0.067346	0.259211

f) Lnpep

LCre	0.05023994	0.7492402	0.140878	20.81
LBmal1	0.588318585	0.7574868	0.031625	5.70
p-value		0.8948006	0.221221	0.462263
LPer1/2	0.403752446	0.6880121	0.030439	12.08
p-value		0.2577693	0.151611	0.66017

g) Nhe3

LCre	0.002133494	2.1961811	0.453673	10.94
LBmal1	0.163101232	1.8351952	0.12892	15.45
p-value		0.0020027	0.039473	0.179934
LPer1/2	0.190640479	1.4543959	0.099464	4.51
p-value		7.31E-08	0.029492	0.084753

h) Nr1h3

LCre	0.001294402	0.5497521	0.123587	15.49
LBmal1	0.017347913	0.3911245	0.098279	22.91
p-value		6.33E-05	0.604091	0.000134
LPer1/2	0.000556986	0.3471626	0.095245	3.20
p-value		2.30E-08	0.463649	1.48E-09

i) Per1

LCre	5.69E-05	0.0354841	0.024971	13.29
LBmal1	7.92E-05	0.0337455	0.015616	14.21
p-value		0.6502389	0.091636	0.410646
LPer1/2	7.21E-07	0.0366388	0.025225	13.11
p-value		0.7676217	0.963322	0.830478

j) Per2

LCre	3.02E-07	0.2783323	0.258662	13.64
LBmal1	1.21E-08	0.3435973	0.205358	13.44
p-value		0.0131065	0.13818	0.744283
LPer1/2	2.12E-09	0.3415696	0.230936	13.36
p-value		0.1614088	0.550532	0.862674

k) Ren1

LCre	0.035732868	0.2794853	0.089088	20.82
LBmal1	0.095183299	0.4215234	0.113539	7.33
p-value		0.0114226	0.745164	0.000961
LPer1/2	0.418496256	0.5373477	0.035376	1.73
p-value		5.28E-07	0.357206	0.306567

Liver

l) Agt

LCre	0.023620585	6.3822008	0.960145	18.43
LBmal1	0.000784676	8.0493419	1.219035	23.48
p-value		4.43E-05	0.604146	0.007755
LPer1/2	0.013269265	6.8811552	1.211975	4.64
p-value		0.2396796	0.671615	3.69E-05

m) Bmal1

LCre	1.01E-08	0.1830218	0.217189	23.72493
LBmal1	6.41E-09	0.0078566	0.00673	23.26666
p-value		9.79E-14	5.64E-12	0.953165
LPer1/2	0.034354199	0.0921598	0.023248	17.87754
p-value		1.73E-06	5.83E-10	0.028166

n) Enpep

LCre	0.023856653	2.0922917	0.326245	16.41
LBmal1	0.001829915	0.9825089	0.212525	0.30
p-value		8.62E-12	0.442907	0.001262
LPer1/2	0.000452061	2.2586104	0.808739	1.67
p-value		0.3059473	0.04091	7.31E-05

o) Lnpep

LCre	0.021349352	0.5133072	0.072404	11.83
LBmal1	0.003724695	0.2884545	0.045635	2.26
p-value		2.85E-11	0.397109	0.00013
LPer1/2	0.008813309	0.4068257	0.062433	4.23
p-value		0.0001649	0.777638	0.000658

p) Per1

LCre	1.74E-06	0.1636768	0.17758	12.79706
LBmal1	1.35E-06	0.0535552	0.042504	13.98803
p-value		4.03E-07	4.38E-06	0.456394
LPer1/2	0.000257939	0.0242357	0.017465	12.08869
p-value		3.02E-09	1.87E-07	0.849424

q) Per2

LCre	4.83E-10	0.3061938	0.243919	13.39755
LBmal1	4.55E-08	0.1487476	0.061326	14.9516
p-value		4.44E-13	5.38E-11	0.07256
LPer1/2	3.50E-06	0.0460165	0.030563	14.4181
p-value		3.00E-19	6.35E-13	0.526313

Lung

r) Ace

LCre	0.001963562	0.3249133	0.203292	19.35543
LBmal1	0.100760957	0.2246243	0.133032	21.83531
p-value		0.1396835	0.458154	0.282955
LPer1/2	0.808403424	1.2547649	0.032879	15.03449
p-value		3.56E-10	0.245521	0.720692

s) Bmal1

LCre	0.000176647	0.0448965	0.038868	23.8986
LBmal1	0.000113206	0.0526955	0.064005	23.08867
p-value		0.457614	0.096656	0.501914
LPer1/2	0.010653123	0.0397713	0.051006	22.90275
p-value		0.708165	0.531617	0.556774

t) Per1

LCre	0.000512567	0.010663	0.005317	14.12175
LBmal1	0.011744216	0.0180117	0.007117	11.44985
p-value		0.0007242	0.519438	0.137287
LPer1/2	0.003917851	0.0545541	0.055299	12.99633
p-value		0.0006393	0.004527	0.89318

Plasma

u) AGT

LCre	0.000244248	71.163665	21.73603	0.433424
LBmal1	0.021422498	51.723954	11.23566	0.456271
p-value		0.0001426	0.106608	0.989416
LPer1/2	0.000140081	23.036444	6.106731	21.5457

p-value		4.61E-15	0.002367	0.191741
v) REN1				
LCre	0.105353575	378.83889	41.85027	4.677158
LBmal1	0.390007083	277.39444	41.02804	6.944744
p-value		0.0101994	0.987566	0.641691
LPer1/2	0.037964313	276.87778	67.77531	22.864
p-value		0.0007504	0.505058	0.055267
w) ACE				
LCre	0.059975914	21.822222	2.341256	3.685668
LBmal1	0.000635239	12.222222	2.753606	7.206776
p-value		2.25E-11	0.756399	0.087674
LPer1/2	0.030495367	15.883333	2.496515	3.408737
p-value		7.47E-06	0.92111	0.91107
x) ALD				
LCre	0.024982002	1392.9575	161.5677	22.74017
LBmal1	0.009255596	1727.3555	617.7133	8.795633
p-value		0.0372016	0.043947	0.011753
LPer1/2	0.013169057	1169.65	220.1517	3.178785
p-value		0.0041303	0.568898	0.043805

Supplemental table S2. List of all statistical parameters and significance values for each gene and plasma RAAS components. The table is divided into two parts: the green box includes the values obtained from a Two-way-ANOVA comparing each genotype with LCre. If the interaction term is significant, then the values for the influence of time or genotype are in (brackets), because their influence is difficult to interpret under those conditions. Finally, the blue box includes the values from a Tukey's post hoc test to correct for multiple testing. *Agt*: Angiotensinogen; *Ace*: Angiotensin I-converting enzyme; *Ren1*: Renin 1; *Nhe3*: Na^{+}/H^{+} exchanger 3; *Agtr1*: Angiotensin II receptor type 1; *Nr1h3*: Liver X receptor α; *Bmal1*: Brain and muscle ARNT-like protein 1; *Per1*: Period 1; *Per2*: Period 2; *Enpep*: Glutamyl aminopeptidase; *Lnpep*: Leucyl and Cystinyl aminopeptidase. * $p < 0.05$; ** $p < 0.01$; *** $p < 0.001$.

	**Two-way ANOVA**			**Tukey's post hoc test**					
	Interaction term	Time	Genotype	ZT0	ZT4	ZT8	ZT12	ZT16	ZT20
**Kidney**									
a)Ace									
LCre	***	(***)	(ns)						
LBmal1				ns	ns	ns	ns	***	ns
LPer1/2				ns	ns	ns	ns	***	ns
LCre	***	(**)	(***)						
LPer1				***	***	***	**	ns	*
LPer2				**	ns	ns	ns	***	**
b) Agt									
LCre	***	(*)	(***)						
LBmal1				***	ns	ns	***	***	ns
LPer1/2				ns	ns	ns	***	***	ns

LCre	**	(*)	(ns)						
LPer1				ns	ns	ns	ns	*	ns
LPer2				ns	ns	ns	ns	ns	ns
c) Agtr1a									
LCre	**	(*)	(**)						
LBmal1				ns	**	***	ns	ns	*
LPer1/2				ns	ns	ns	ns	ns	ns
LCre	**	(ns)	(***)						
LPer1				ns	ns	ns	ns	ns	***
LPer2				ns	ns	ns	ns	**	ns
d) Bmal1									
LCre	ns	***	ns						
LBmal1				ns	ns	ns	ns	ns	ns
LPer1/2				ns	ns	ns	ns	ns	ns
LCre	ns	***	ns						
LPer1				ns	ns	ns	ns	*	ns
LPer2				ns	ns	ns	ns	ns	ns
e) Enpep									
LCre	**	(ns)	(***)						
LBmal1				***	ns	ns	ns	***	ns
LPer1/2				**	ns	ns	ns	***	ns
LCre	ns	***	**						
LPer1				ns	ns	ns	ns	ns	ns
LPer2				ns	ns	ns	ns	ns	ns
f) Lnpep									

LCre	**	(ns)	(ns)						
LBmal1				ns	ns	ns	ns	*	**
LPer1/2				ns	ns	ns	ns	ns	ns
LCre	ns	***	ns						
LPer1				ns	ns	ns	ns	ns	ns
LPer2				ns	ns	ns	ns	ns	ns
g) Nhe3									
LCre	*	(ns)	(***)						
LBmal1				ns	ns	*	**	*	ns
LPer1/2				ns	ns	***	***	**	ns
LCre	***	(***)	(***)						
LPer1				ns	ns	***	***	ns	ns
LPer2				ns	ns	ns	ns	ns	ns
h) Nr1h3									
LCre	***	(ns)	(***)						
LBmal1				ns	ns	ns	***	***	ns
LPer1/2				ns	ns	ns	***	***	***
LCre	ns	ns	***						
LPer1				ns	**	ns	ns	ns	**
LPer2				ns	ns	ns	ns	ns	ns
i) Per1									
LCre	ns	***	ns						
LBmal1				ns	ns	ns	ns	ns	ns
LPer1/2				ns	ns	ns	ns	ns	ns
LCre	ns	***	ns						
LPer1				ns	ns	ns	ns	ns	ns

LPer2				ns	ns	ns	ns	ns	ns
j) Per2									
LCre	ns	***	*						
LBmal1				ns	ns	ns	ns	ns	ns
LPer1/2				ns	ns	ns	ns	ns	ns
LCre	ns	***	ns						
LPer1				ns	ns	ns	ns	ns	ns
LPer2				ns	ns	ns	ns	ns	ns
k) Ren1									
LCre	***	(*)	(***)						
LBmal1				ns	ns	***	ns	ns	ns
LPer1/2				**	**	***	***	ns	**
LCre	ns	ns	***						
LPer1				***	***	***	***	**	***
LPer2				ns	ns	ns	ns	ns	ns
**Liver**									
l) Agt									
LCre	**	(ns)	(***)						
LBmal1				ns	**	*	ns	ns	**
LPer1/2				ns	ns	***	ns	ns	ns
LCre	ns	**	ns						
LPer1				ns	ns	ns	ns	ns	ns
LPer2				ns	ns	ns	ns	ns	ns

m) Bmal1									
LCre	***	(***)	(***)						
LBmal1				***	***	ns	ns	**	***
LPer1/2				***	***	ns	***	ns	***
LCre	ns	***	ns						
LPer1				ns	ns	ns	ns	ns	ns
LPer2				ns	ns	ns	ns	ns	ns
n) Enpep									
LCre	***	(**)	(***)						
LBmal1				**	*	*	***	***	*
LPer1/2				**	**	ns	ns	**	ns
LCre	ns	***	ns						
LPer1				ns	ns	ns	ns	***	ns
LPer2				ns	*	ns	ns	ns	ns
o) Lnpep									
LCre	***	(***)	(***)						
LBmal1				***	**	***	***	***	*
LPer1/2				*	ns	ns	***	***	ns
LCre	ns	**	ns						
LPer1				ns	ns	ns	ns	ns	ns
LPer2				ns	ns	ns	ns	ns	ns
p) Per1									
LCre	***	(***)	(***)						
LBmal1				ns	ns	***	***	***	***
LPer1/2				ns	ns	***	***	***	***
LCre	***	(***)	(***)						

LPer1				ns	ns	***	***	***	***
LPer2				ns	ns	ns	*	***	*
q) Per2									
LCre	***	(***)	(***)						
LBmal1				ns	***	***	***	***	**
LPer1/2				***	***	***	***	***	***
LCre	***	(***)	(***)						
LPer1				ns	ns	ns	***	***	ns
LPer2				**	***	***	***	***	***
**Lung**									
r) Ace									
LCre	ns	ns	***						
LBmal1				ns	ns	ns	ns	ns	ns
LPer1/2				*	***	***	**	***	**
s) Bmal1									
LCre	ns	***	ns						
LBmal1				ns	ns	ns	ns	ns	ns
LPer1/2				ns	ns	ns	ns	ns	ns
t) Per1									
LCre	**	(***)	(***)						
LBmal1				ns	ns	ns	ns	ns	ns
LPer1/2				ns	ns	ns	***	*	ns
**Plasma**									

u) AGT									
LCre	***	(***)	(***)						
LBmal1				***	*	**	ns	*	**
LPer1/2				***	***	***	***	***	***
v) REN									
LCre	ns	ns	**						
LBmal1				ns	*	ns	ns	ns	*
LPer1/2				ns	ns	*	ns	ns	ns
w) ACE									
LCre	ns	***	***						
LBmal1				***	***	**	***	***	***
LPer1/2				*	ns	***	**	ns	***
x) ALD									
LCre	***	(***)	(***)						
LBmal1				ns	ns	***	ns	ns	ns
LPer1/2				ns	ns	ns	ns	ns	ns

Supplemental table 3. List of primer sequences and TaqMan probes for RT-PCR

Gene Name	All sequences in 5' to 3' direction (FW: forward, RV: reverse, TM: TaqMan probe, FAM: 6-fluorescine, BHQ1: black hole quencher 1
Nono	FW: TCT TTT CTC GGG ACG GTG GAG<br>RV: GTC TGC CTC GCA GTC CTC ACT<br>TM: FAM-CGT GCA GCG TCG CCC ATA CTC CGA GC-BHQ1
Agt	FW: CGT TCA CTT CCA AGG AAC GAT G<br>RV: GCT GTT GTC CAC CCA GAA TT<br>TM: FAM-TTC TCT CAG CTG CCT GGA GTC CAT G-BHQ1
Ace	FW: GGT CCG AGT ACA TCA ACC TGG A<br>RV: GCG CCC ACA TGT TCC CTA G<br>TM: FAM-TGG CCC CAT TCC TGC CCA TCT G-BHQ1
Ren1	FW: CTC TCT GGG CAC TCT TGT TGC<br>RV: GCA TTT TCT TGA GCG GGA TTC G<br>TM: FAM-CTG CAC CTT CAG TCT CCC AAC ACG CAC C-BHQ1
Agtr1	FW: GAC AGG GTG GAG GCC CTC AT<br>RV: GGA TTT CCA GCT GGG GCA GAG T<br>TM: FAM-CAT GCG GGA GCT GAG TAA GCT GA-BHQ1
Nr1h3	FW: AGG GAT AGG GTT GGA GTC AGC<br>RV: TTT GTG GAC GAA GCT CTG TCG<br>TM: FAM-AGC CTA CAG CCC TGC TCC CCA GG-BHQ1
Slc9a3	FW: CAT GCG GGA GCT GAG TAA GCT GA<br>RV: TTG TCC GTA CTT GGG GAG CGA<br>TM: FAM-CGC CGT GGG TCC CTG GCC TTC A-BHQ1
Bmal1	FW: GCA ATG CAA TGT CCA GGA AG<br>RV: GCT TCT GTG TAT GGG TTG GT<br>TM: FAM-ACC GTG CTA AGG ATG GCT GTT CAG CA-BHQ1
Per1	FW: GGC ATG ATG CTG CTG ACC ACG<br>RV: ACT GGG GCC ACC TCC AGT TC<br>TM: FAM-TGG CCC TCC CTC ACC TTA GCC TGT TCC T-BHQ1
Enpep	FW: GTG CAT CGG TTC ACT GCT AT<br>RV: CTT CTG ATT GGG CTG GAC AT<br>TM: FAM-CCA GGA GCG GCA AAC CAC TCA-BHQ1
Anpep	FW: TCA CAC TCA TCT ACC CCA ACA<br>RV: CTC AGT CAT GGT GCA GGA AG<br>TM: FAM-TGC TTC CCA AAG AGT CCA AGC CCT-BHQ1
Rnpep	FW: GCT TTG GCC ATT GGA GAT CT<br>RV: TCC TTG GCA GCT TCA ATC AG<br>TM: FAM-CCC AGG AGC CGG GTG TGG G-BHQ1
Lnpep	FW: ATG CCA AAG GTG GCT TGT AT<br>RV: ATG CCA AAG GTG GCT TGT AT<br>TM: FAM-CGC ATG CAA CCA GAA AGT CAG CC-BHQ1
Dpp3	FW: CAA AGG CCC CAT TGT AGA GA<br>RV: CCA CGA AGC CTT CAA ACT CT<br>TM: FAM-CCG TGA CCC CTT TGG CTC CCG-BHQ1
Sirt2	FW: CAG GCC AGA CGG ACC CCT TC<br>RV: AGG CCA CGT CCC TGT AAG CC<br>TM: FAM-TGA TGG GCC TGG GAG GTG GCA TGG A-BHQ1

Cdk5	FW: GGT GAA CGT CGT GCC CAA GC<br>RV: TGA GAT GCG CTG CAC AGG GT<br>TM: FAM-TGC CAC GGG GAG GGA CCT GTT GCA-BHQ1
Wdr5	FW: CCA CAC AGA GCA AGC CCA C<br>RV: CAC AGA GGA CAC AGC TTT GGT GT<br>TM: FAM-GCC CTG AAG TTC ACC CTG GCT GGC C-BHQ1
Atp5h	FW: TGC CCT GAA GAT TCC TGT GCC T<br>RV: ACT CAG CAC AGC TCT TCA CAT CCT<br>TM: FAM-TCT CCT CCT GGT CCA CCA GGG CTG TGT-BHQ1
