# Supplementary material for: Loss of the liver circadian clock affects the expression of intrarenal renin-angiotensin system components
Source: Sci Rep. 2025 Dec 29;16:4158. doi: 10.1038/s41598-025-34303-w (PMC12859090; doi:10.1038/s41598-025-34303-w)
Supplement: Supplementary file 7 — Supplementary Material 7 [file 41598_2025_34303_MOESM7_ESM.pdf]

Hassan et al., Loss of the Liver Circadian Clock Affects the Expression of Intrarenal Renin-Angiotensin System Components

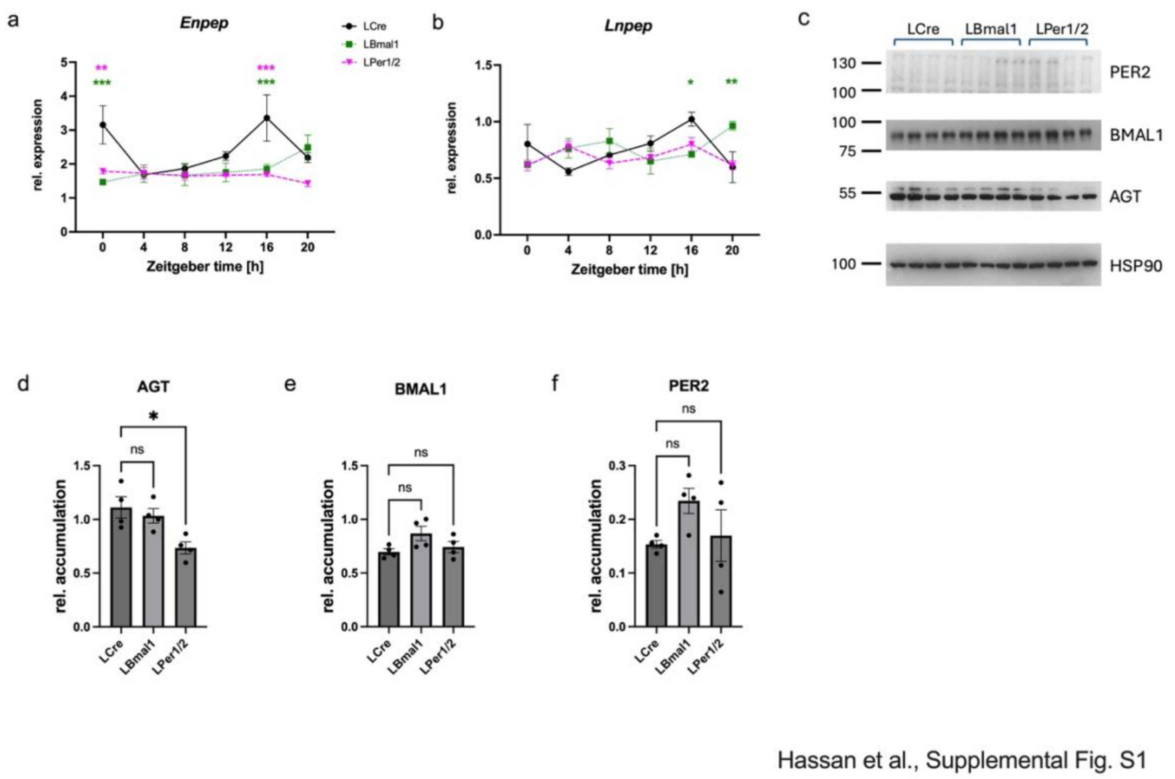

Supplemental Fig. S1. Effect of targeted deletion of hepatic circadian clock genes on *Enpep* and *Lnpep* and clock protein accumulation in kidney. Relative expression of (a) *Enpep* and (b) *Lnpep* in LCre (black solid lines), LBmal1 (green dotted lines) and LPer1/2 (magenta dashed lines) mice at six different *zeitgeber* time points with 4-hour intervals. Green stars indicate significant differences between LCre and LBmal1, and magenta stars indicate significant differences between LCre and LPer1/2. Data are presented as mean ± SEM (n = 3). (c) western blot analysis of the indicated proteins at ZT8 and quantification (n = 4) (d-f). Note that for PER2 we quantified the same region of the gel as in Fig. 1g. The band is barely visible, because ZT8 is the time of lowest accumulation of PER2 during the circadian cycle. On the left are the positions of size markers [kDa]. *Enpep*: Glutamyl aminopeptidase; *Lnpep*: Leucyl and Cystinyl aminopeptidase; BMAL1: Brain and muscle ARNT-like 1; PER2: PERIOD 2; HSP90: heat shock protein 90. Two-way ANOVA with Tukey's post hoc test (a,b) and one-way ANOVA (d-f), * $p < 0.05$; ** $p < 0.01$; *** $p < 0.001$.

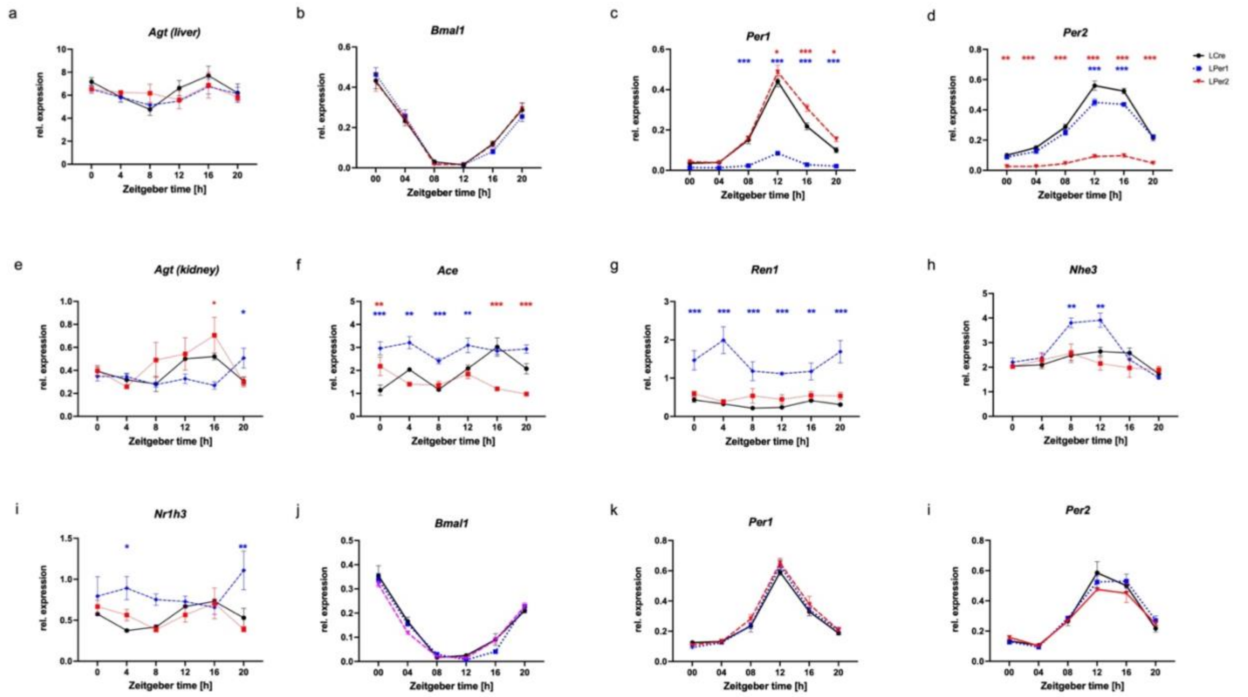

Hassan et al., Supplemental Fig. S2

Supplemental Fig. S2. Effect of targeted deletion of hepatic *Per1* or *Per2* on liver *Agt* and irRAS components. Relative expression of (a) liver *Agt*, (b) liver *Bmal1*, (c) liver *Per1*, (d) liver *Per2*, (e) kidney *Agt*, (f) kidney *Ace*, (g) kidney *Ren1*, (h) kidney *Nhe3*, (i) kidney *Nr1h3*, (j) kidney *Bmal1*, (k) kidney *Per1*, and (l) kidney *Per2* in LCre (black solid lines), LPer1 (blue dashed lines) and LPer2 (red dotted lines) mice at six different *zeitgeber* time points with 4-hour intervals. Blue stars indicate significant differences between LCre and LPer1 and red stars indicate significant differences between LCre and LPer2. Data are presented as mean ± SEM (n = 3). *Agt*: Angiotensinogen; *Ace*: Angiotensin I-converting enzyme; *Ren1*: Renin 1; *Nhe3*: Na^+/H^+ exchanger 3; *Agtr1*: Angiotensin II receptor type 1; *Nr1h3*: Liver X receptor α. Two-way ANOVA with Tukey's post hoc test, * $p < 0.05$; ** $p < 0.01$; *** $p < 0.001$.

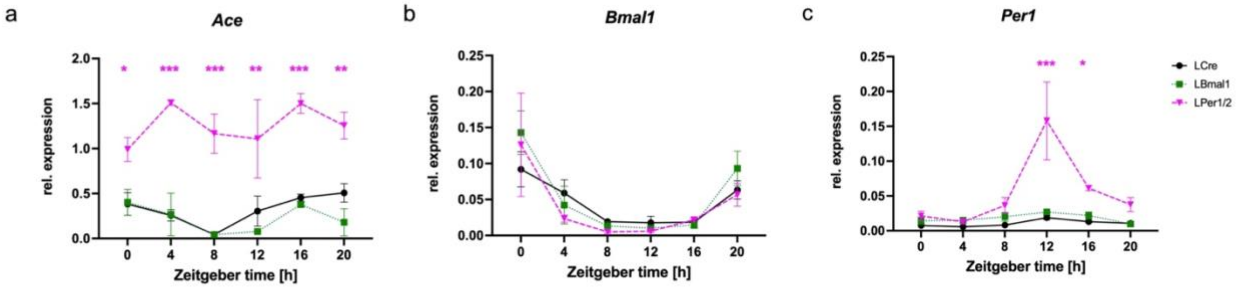

Hassan et al. Supplemental Fig. S3

Supplemental Fig. S3. Effect of targeted deletion of hepatic circadian clock genes on lung *Ace* and clock genes. Relative expression of (a) *Ace*, (b) *Bmal1*, and (c) *Per1* in LCre (black solid lines), LBmal1 (green dotted lines) and LPer1/2 (magenta dashed lines) mice at six different *zeitgeber* time points with 4-hour intervals. Green stars indicate significant differences between LCre and LBmal1, and magenta stars indicate significant differences between LCre and LPer1/2. Data are presented as mean ± SEM (n = 3). *Ace*: Angiotensin I-converting enzyme; *Bmal1*: Brain and muscle ARNT-like protein 1; *Per1*: Period 1. Two-way ANOVA with Tukey's post hoc test, * $p < 0.05$; ** $p < 0.01$; *** $p < 0.001$.

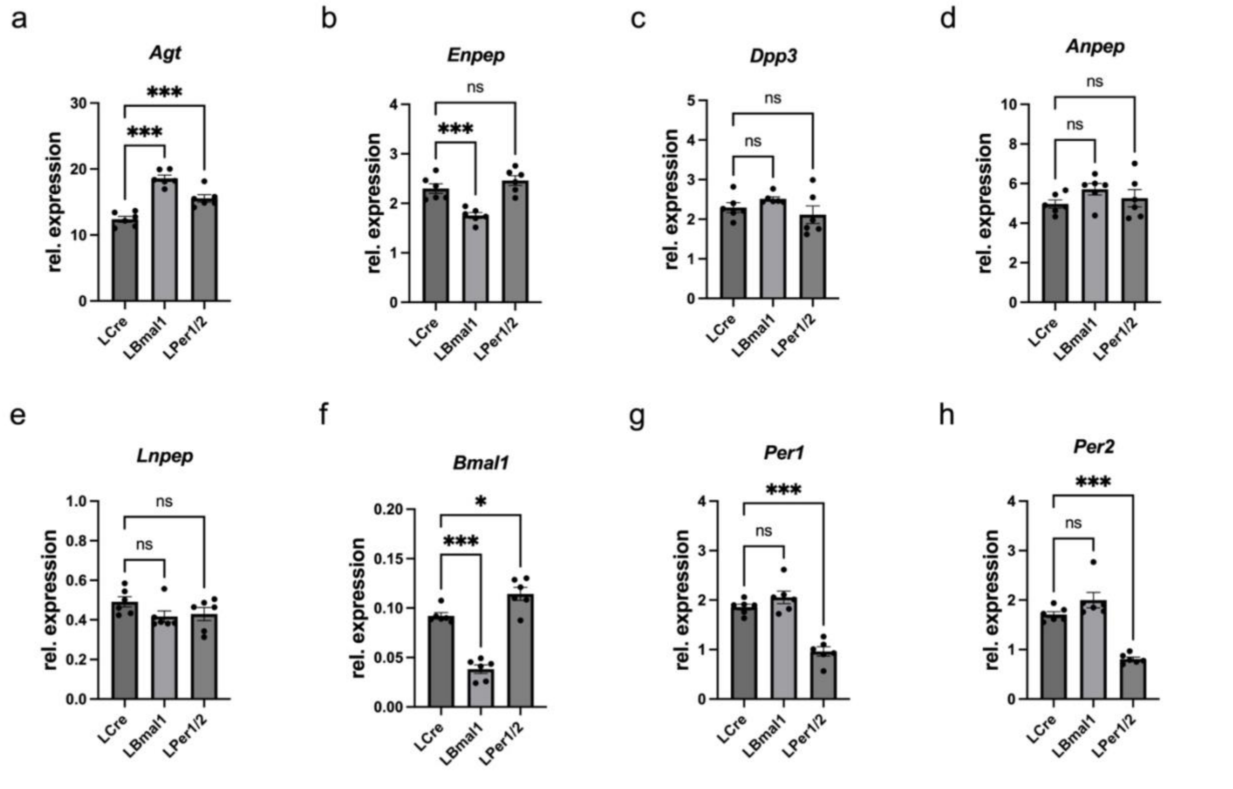

Hassan et al. Supplemental Fig. S4

Supplemental Fig. S4. Effect of targeted deletion of hepatic circadian clock genes on liver gene expression at ZT8. Relative expression of (a) *Agt*, (b) *Enpep*, (c) *Dpp3*, (d) *Anpep*, (e) *Lnpep*, (f) *Bmal1*, (g) *Per1*, and (h) *Per2* in LCre, LBmal1 and LPer1/2 mice at ZT8. Data are presented as mean ± SEM (n = 6 each). *Agt*: Angiotensinogen; *Enpep*: Glutamyl aminopeptidase; *Dpp3*: Dipeptidyl peptidase 3; *Anpep*: Alanyl aminopeptidase; *Lnpep*: Leucyl and Cystinyl aminopeptidase; *Bmal1*: Brain and muscle ARNT-like protein 1; *Per1*: Period 1; *Per2*: Period 2. One-way ANOVA with Dunnett's post hoc test compared to LCre, ns: non-significant; ** $p < 0.01$; *** $p < 0.001$.
